# Adipocytes-induced ANGPTL4/KLF4 axis drives glycolysis and metastasis in triple-negative breast cancer

**DOI:** 10.1186/s13046-025-03458-9

**Published:** 2025-07-04

**Authors:** Dou Yin, Nana Fang, Yaling Zhu, Xiaoqing Bao, Juan Yang, Qingyu Zhang, Ruimeng Wang, Jiahui Huang, Qibing Wu, Fang Ma, Xiaohui Wei

**Affiliations:** 1 Department of Pathophysiology, School of Basic Medical Sciences, Anhui Medical University, Hefei, 230032 China; 2 Department of Oncology, the First Affiliated Hospital of Anhui Medical University, Hefei, 230001 China; 3 Second Clinical Medical College, Anhui Medical University, Hefei, 230032 China; 4 Center for Scientific Research of Anhui Medical University, Hefei, 230032 China

**Keywords:** Adipocytes, Lipolysis, ANGPTL4, KLF4, Triple-Negative Breast Cancer

## Abstract

**Background:**

The adipocyte-rich tumor microenvironment (TME) is recognized as a key factor in promoting cancer progression. A distinct characteristic of peritumoral adipocytes is their reduced lipid content and the acquisition of a proinflammatory phenotype. However, the underlying mechanisms by which adipocytes rewire metabolism and boost tumor progression in triple-negative breast cancer (TNBC) remain poorly understood.

**Methods:**

We utilized transcriptomic analysis, bioinformatic analysis, metabolic flux analysis, protein-protein docking, gene and protein expression profiling, in vivo metastasis analysis and breast cancer specimens to explore how adipocytes reprogram tumor metabolism and progression in TNBC.

**Results:**

Our findings reveal that Angiopoietin-like 4 (ANGPTL4) exhibits significantly higher expression levels in adipocyte-rich tumor circumstance compared to the symbiotic environment lacking of adipocyte. Furthermore, ANGPTL4 expression in tumor cells is essential for adipocyte-driven glycolysis and metastasis. Interleukin 6 (IL-6), enriched in cancer-associated adipocytes, and lipolysis-derived free fatty acids (FFAs) released from adipocytes, amplify ANGPTL4-mediated glycolysis and metastasis through activation of STAT3 and PPARα pathways in TNBC cells. Additionally, ANGPTL4 interacts with transcription factor KLF4 and enhances KLF4 activity, which further drives glycolysis and metastasis, whereas KLF4 knockdown attenuates migration and glycolysis in TNBC cells. Importantly, Elevated ANGPTL4 and KLF4 expression was observed in metastatic breast cancer specimens compared to non-metastatic cases and was positively correlated with poor prognosis.

**Conclusion:**

Collectively, our results uncover a complex metabolic interaction between adipocytes and TNBC cells that promotes tumor aggressiveness. ANGPTL4 emerges as a key mediator in this process, making it a promising therapeutic target to inhibit TNBC progression.

**Supplementary Information:**

The online version contains supplementary material available at 10.1186/s13046-025-03458-9.

## Introduction

Breast cancer is a highly heterogeneous disease, categorized into four subtypes based on hormone receptor and human epidermal growth factor receptor 2 (HER2) status: luminal A (HR+/HER2-), luminal B (HR+/HER2+), HER2-positive (HR-/HER2+), and triple-negative breast cancer (TNBC, HR-/HER2-) [1]. TNBC, representing approximately 12–17% of breast cancer cases, is distinguished by its aggressive clinical behavior, high metastatic potential, and limited therapeutic options, contributing to its poor prognosis [2, 3].

Metabolic alterations interact to shape the fate of cancer and stromal cells within the tumor microenvironment, with metabolic reprogramming serving as a hallmark of tumor adaptation [4, 5]. Cancer cells drive the metabolic heterogeneity of breast cancer, with TNBC exhibiting distinct metabolic phenotypes and more pronounced dysregulation compared to luminal or HER2-enriched subtypes [6]. Multi-omics analyses reveal that TNBC has an elevated glycolytic flux associated with poorer outcomes [6–8]. The metabolic heterogeneity of breast cancer arises from multiple factors, including oncogenic pathways such as PI3K, STAT3, MYC, and Hippo signaling, which contribute to TNBC’s glycolytic preference [7, 9, 10]. Additionally, recent studies emphasize the critical role of bidirectional interactions between cancer cells and the TME in promoting the dysregulated synthesis and transport of metabolic intermediates, including lactate, glutamine, and fatty acids, which fuel cancer cell energy production and aggressive behavior [11–15]. Consequently, components of the tumor microenvironment may significantly influence tumor metabolism, steering cancer cells toward the preferential utilization of specific metabolic pathways.

Adipocytes, a prominent component of the adipose-enriched tumor microenvironment, contribute to cancer progression through the secretion of cytokines (e.g., leptin, interleukin-6), extracellular vesicles, providing lipids and the extracellular matrix remodeling [16–21]. Cancer cells induce adipocyte dedifferentiation, pro-inflammatory cytokine secretion, and free fatty acid (FFA) production, facilitating metabolic crosstalk [17]. Tumors actively stimulate adipocyte lipolysis via the adipose triglyceride lipase (ATGL)-dependent pathway, leading to FFA release [22], which is transferred to cancer cells via CD36 or FATPs to fuel fatty acid oxidation (FAO) [23, 24], enhancing tumor aggressiveness. Adipocyte-derived metabolites, such as β-hydroxybutyrate [25], creatine [26] and glutathione (GSH) [27], further support tumor growth and metastasis by activating oncogenic pathways. Beyond FAO activation, adipocytes also reprogram glucose metabolism in cancer cells, directing glucose flux toward glycerol-3-phosphate (G3P) synthesis [28]. Additionally, adipocytes enhance glycolytic ATP and lactate production in cancer cells, promoting a shift toward aerobic glycolysis [22]. While adipocytes significantly influence cancer cell metabolism, the precise molecular mechanisms governing this reciprocal metabolic interaction remain unclear. Understanding the critical pathways mediating the interplay between cancer cells and the CAAs will unveil novel therapeutic targets for the treatment of breast cancer.

We investigate the role of adipocytes in TNBC cell metabolism and progression, demonstrating that adipocytes provide fatty acids and IL-6 to fuel glycolysis and metastasis in TNBC cells. These adipocyte-derived factors activate ANGPTL4 through PPARα and STAT3 pathways, which, in turn, reprogram TNBC metabolism and metastasis by regulating the transcription factor KLF4. Our findings uncover a novel glycolytic pathway that mediates reciprocal between adipocytes and TNBC cells within the tumor microenvironment.

## Methods and materials

### Cell culture and reagents

Breast cancer cell lines were obtained from the Chinese Academy of Sciences (Shanghai, China). MDA-MB-231, MDA-MB-468 and SK-BR-3 cells were cultured in DMEM (GIBCO). BT474 cells were cultured in RPMI-1640(GIBCO). MCF7 cells were cultured in Eagle’s Minimum Essential Medium supplement with insulin(0.01 mg/mL). Murine 3T3-L1 preadipocytes were acquired from Meisen CTCC. Human preadipocytes were obtained from ScienCell Research Laboratories and cultured as previously reported [20]. Recombinant human IL-6 (R&D System) was utilized at a final concentration of 5 ng/mL. The JAK inhibitor Ruxolitinib was procured from SelleckChem, while the PPARα inhibitor MK-886 was obtained from MedChemExpress. Palmitic acid was purchased from Merck.

### Differentiation into adipocytes

The differentiation of 3T3-L1 cells into adipocytes was carried out as previously described [24]. Briefly, cells were cultured in DMEM until reaching full confluency, and differentiation was induced 2 days post-confluency by replacing the culture medium with differentiation medium consisted of 500µM isobutyl-1-methylxanthine (IBMX), 1 µM dexamethasone, and 10 µg/mL insulin (all from Merck) for three days. Thereafter, cells were cultured in a differentiation medium supplemented with 10 µg/mL insulin for an additional 4 days. Subsequently, cells were cultured in completed DMEM medium until they fully differentiated into mature adipocytes characterized by lipid droplet accumulation. Human preadipocytes were differentiated into mature adipocytes using Differentiation Medium (PADM, Cat. #7221), followed by maintenance in Adipocyte Medium (AdM, Cat. #7201) to sustain the mature adipocyte phenotype.

### Coculture and migration assays

Human adipocytes or 3T3-L1 adipocytes were co-cultured with cancer cells in a Transwell system (0.4 μm, Corning) to enable indirect cellular interaction as previously indicated. Breast cancer cells were seeded in the upper compartment, while adipocytes were cultured in the lower compartment. To evaluate the influence of adipocytes on cancer cell migration, tumor cells were harvested following a three-day co-culture and subjected to transwell migration assay with 8 μm pore-sized inserts (BD Biosciences, USA). A total of 5 × 10⁴ to 8 × 10⁴ breast cancer cells were plated in the upper chamber with medium containing 2% FBS, allowing migration towards the lower chamber containing 10% FBS as a chemoattractant. Migration assays were also performed using tumor cells cultured alone as controls. After 18 ~ 24 h, the migrated cells were fixed, stained using the Diff-Quik Stain Kit (Jiancheng), imaged, and subsequently quantified with ImageJ software.

### Hanging drop invasion

Multicellular aggregates were established employing the hanging drop method. Briefly, breast cancer cells, either co-cultured with adipocytes or maintained as monocultures for three days, were resuspended in DMEM containing 20% methylcellulose (Sigma) and 10%Matrigel (Corning). Droplets (25 µL) containing approximately 1,000 cells were incubated for 48 h to facilitate multicellular aggregation. For the three-dimensional (3D) Matrigel invasion assay, the aggregates were rinsed with culture medium and subsequently plated onto a Matrigel-coated surface. Invasive potential was assessed 24 h post-seeding, with medium replenishment every two days. The invasion assay was conducted for up to seven days to allow tumor cell invasion to become evident.

### 2-NBDG glucose uptake assay

Following a three-day co-culture period with adipocytes, breast cancer cells were harvested and subjected to serum starvation for 30 min. Glucose uptake was then assessed using 2-NBD-Glucose (100 µM, ThermoFisher) as a fluorescent tracer for an additional 30 min. The intracellular fluorescence intensity, indicative of glucose uptake, was subsequently analyzed by CytoFlex (Beckman).

### ATP measurement

Breast tumor cells cultured either with or without adipocytes for 72 h, were plated into 96-well plate (2000 cell/well). After 16 h of incubation, ATP levels were quantified using the Cell-Titer-Glo assay kit (Promega) following the manufacturer’s protocol. Luminescence signals were measured using a plate reader (Enspire, PE) to assess cellular energy metabolism.

### Bodipy 493/503 fluorescence staining

Adipocytes and breast cancer cells subjected to the indicated treatments were stained with Bodipy 493/503 (Thermo Fisher Scientific) at a concentration of 2 µg/mL. Fluorescence imaging was performed using ZEISS LSM880 confocal microscope to visualize lipid accumulation within the cells.

### RNA-sequencing analysis

Total RNA of cocultured human TNBC(MDA-MB-468) and non-TNBC(BT-474) breast cancer cells or monoculture controls were extracted using TRIzol reagent (Vazyme, Nanjing, China) following the manufacturer’s protocol. Complementary DNA (cDNA) libraries were subsequently constructed from the extracted RNA using the NEBNext Ultra Directional RNA Library Prep Kit for Illumina^®^ (NEB, USA). Paired-end sequencing was performed by Novogene (Beijing, China) on the Illumina NovaSeq 6000 platform (Illumina, USA). RNA sequencing (RNA-seq) reads were aligned to the human reference genome (GRCh38) using STAR (version 2.5.3a), and read counts were quantified using Feature Counts software. Gene expression levels were normalized using the fragments per kilobase of transcript per million mapped reads (FPKM) algorithm. Differentially expressed genes (DEGs) were identified using the DESeq2 package, with the significance threshold set at |log2(fold change)| ≥ 1 and p-value < 0.05. The RNA-seq dataset has been deposited in GEO database under accession number GSE287031.

### Oxygen consumption and extracellular acidification rate measurements

The impact of adipocytes and ANGPTL4 on glycolysis and mitochondrial metabolism was assessed with Seahorse XFe96 Analyzer (Agilent) by measuring the extracellular acidification rate (ECAR) and oxygen consumption rate (OCR). Briefly, 1.5 × 10⁴ to 2 × 10⁴ breast cancer cells, with or without co-culture with adipocytes, were plated per well into Seahorse plates and incubated overnight for cell adhesion. Following washing, the cells were maintained in Seahorse base medium (Agilent) enriched with glucose (25 mM, Sigma-Aldrich), sodium pyruvate (1 mM, Corning), and glutamine (2 mM, Corning) to facilitate subsequent assessment of oxygen consumption rate (OCR) and extracellular acidification rate (ECAR). The assay medium was adjusted to a pH of 7.4, and cells were incubated in a non-CO_2_ incubator for 1 h prior to initiating the assay. Sequential injections of oligomycin (1 µM, Agilent), FCCP (2 µM, Agilent), antimycin A (1 µM, Agilent), and rotenone (1 µM, Agilent) were administered. To ensure precision in data interpretation, oxygen consumption rate (OCR) and extracellular acidification rate (ECAR) values were normalized relative to total protein content.

### Transient transfection with SiRNAs

Small interfering RNA (siRNA) targeting ANGPTL4 was synthesized by GenePharma (Shanghai, China). Specifically, siANGPTL4#1 (5’-CACCAUGUUGAUCCAGCCCAU-3’) and siANGPTL4#2 (5’-CUGCGAAUUCAGCAUCUGCAA-3’) were used to knock down ANGPTL4 expression in breast cancer cells, while a non-targeting siRNA sequence (Negative Control, 5’-UUCUCCGAACGUGUCACGUTT-3’) was utilized as a control. The siRNA and corresponding negative control were diluted in serum-free medium and transfected using Lipofectamine3000 (Thermo Fisher Scientific). To knock down KLF4 expression, KLF4-specific siRNAs (KLF4#1, 5’-GGAGAGAGACCGAGGAGUUTT-3’; KLF4#2, 5’-CAGAGGAGCCCAAGCCAAATT-3’) were employed. Following 8 h of incubation, the cells were cultured in complete medium for 48–72 h, and subsequently, the cells were collected for qRT-PCR, Western blot analysis, co-culture experiments, or transwell migration assays.

### Transfection and generation of stable cells

shRNAs targeting human ANGPTL4, along with a negative control shRNA, were obtained from GenePharma. To generate stable ANGPTL4-overexpressing cells, a full-length human ANGPTL4 gene lentiviral vector was acquired from GenePharma. Lentiviral particles were produced by transfecting 293T cells with shRNA constructs or ANGPTL4 plasmid alongside the pMD2.G and psPAX2 plasmids. Following a 48-hour incubation, the conditioned medium containing viral particles was harvested and utilized to transfect target cells for 72 h. Stable cell lines were subsequently generated by selecting for puromycin resistance at a concentration of 10 µg/mL. After 2 to 3 passages in the presence of puromycin, the stable cell lines were used for further experimental analysis.

### Co-immunoprecipitation (Co‑IP)

For immunoprecipitation, briefly, cells were lysed with NP-40 buffer (20 mM Tris-HCl, pH 7.5, 150 mM NaCl, 1.5 mM MgCl_2_, 2 mM EDTA, 0.5% NP-40) supplemented with protease inhibitor cocktail (Beyotime, China) for 60 min on ice. Cell lysates were incubated with primary antibodies in a rotating incubator overnight at 4 °C, followed by incubation with protein A/G agarose (MedChemExpress, MCE) beads for another 6 h. The immunoprecipitates were washed seven times with lysis buffer (Beyotime, China) and analyzed by immunoblots. Specifically, secondary antibodies (HRP Conjugated Anti-rabbit IgG for IP Nano-secondary antibody ( NBI01H, HUABIO) or HRP Conjugated Anti-mouse IgG for IP Nano-secondary antibody(NBI02H, HUABIO)) were applied to avoid visualizing IgG heavy chain and light chain signals.

### Extraction of nuclear and cytoplasm protein and Western blot assay

Cells were collected and centrifuged at 1000×g for 5 min at 4 °C, and the cell pellets were collected. Cytoplasmic fractions from cells were lysed with buffer (20 mM Tris-HCl, pH7.4, 10mM NaCl, 3mM MgCl_2,_ 0.5% NP-40) supplemented with protease inhibitor cocktail. Nuclear fractions from cells were lysed with RIPA buffer supplemented with protease inhibitor cocktail, and then the nuclear lysates were sonicated using an Ultrasonic Cell Disruptor (Scientz). Equal amounts of proteins were fractionated by 6–12% SDS–polyacrylamide gel electrophoresis (SDS–PAGE). Signals were detected using Western ECL Substrate (Epyzme, China). Primary antibodies against the following proteins were used: ANGPTL4 (Abcam, ab196746), PFKP (Cell Signaling, 8164), KLF4 (MA5-15672, Thermo Fisher). PGK1(17811-1-AP), HK2 (15010-1-AP), PGAM1 (16126-1-AP), PKM2 (15822-1-AP), LDHA (19987-1-AP), β-actin(20536-1-AP) were purchased from Proteintech. PPARA (A24835,), Phospho-STAT3-Y705(AP0705), STAT3(A11216), were purchased form Abclonal. Flag(M1403-2), HA (HA721750), Histone H3( M1309-1) were purchased from HUABIO.

### Tail vein metastasis assay

Female NOD/SCID mice aged 6–8 weeks were obtained from GemPharmatech (Nanjing, China). Triple-negative breast cancer MDA-MB-231 cells (5 × 10^5 cells per mouse) were administered via tail vein injection. Lung metastasis was monitored 21 days post-injection using bioluminescence imaging with an IVIS spectrum in vivo animal imaging system (Xenogen). Following the imaging, the mice were euthanized, and lung tissues were excised for further analysis. Metastatic tumor burden in the lungs was evaluated through formalin fixation and hematoxylin and eosin (H&E) staining. To explore the role of ANGPTL4-KLF4 signaling in adipocyte-enriched TNBC progression, MDA-MB-231 cells were monocultured, cocultured with adipocytes, or cocultured with adipocytes following ANGPTL4 or KLF4 depletion. After 3 days, these cells were harvested, centrifuged, and gently resuspended in serum free media to a concentration of 5 × 10^5 cells per 200µL. A total of 5 × 10^5^ cells were injected into the tail vein of each Balb/c mouse. After 14 days, mice were sacrificed and lungs were isolated and sectioned for histological studies by fluorescence microscopy. The rest of the lungs were formalin-fixed and paraffin embedded for hematoxylin and eosin staining. All animal procedures in this study were performed in compliance with the guidelines established by the Institutional Animal Care and Use Committee and were approved by the Institutional Animal Care and Ethics Committee of Anhui Medical University.

### Patient breast cancer tissue microarray and immunohistochemistry

A human breast cancer tissue microarray (HBreD122-Su-01), containing 58 breast cancer tissues and 49 adjacent breast tissues, was procured from Shanghai Outdo Biotech Co., Ltd. (China). All tissues were formalin-fixed, paraffin-embedded, and subjected to immunohistochemical (IHC) analysis to evaluate ANGPTL4 expression. IHC staining was conducted using an anti-ANGPTL4(ab196746, Abcam) or anti-KLF4 (MA5-15672, Thermo Fisher) antibody and the Dako Cytomation EnVision System-HRP (DAB) detection kit. Stained images were acquired with a microscope, and expression levels were quantified using a modified histochemical score (MH-score, MHs), which incorporated both staining intensity and the proportion of positively stained cells. For prognostic analysis, samples were categorized as “low expression” (MHs < 10) or “high expression” (MHs ≥ 10).

### Molecular docking analysis

The three-dimensional structures of ANGPTL4 (PDB: 6u1u), KLF4 (PDB: 6vtx), and KLF5 (PDB: 2ebt) were acquired from the Protein Data Bank (PDB). Protein-protein docking simulations were performed using Discovery Studio to predict the binding interactions between ANGPTL4 and KLF4 or KLF5. The resulting complexes were analyzed using RDOCK, and the most plausible interactions were visualized and mapped using PyMOL.

### Bioinformatics analysis

Gene expression profiling data from the TCGA database and the GEO datasets GSE65194 and GSE76124 were analyzed to evaluate gene expression patterns in human breast cancer. The association between gene expression and survival in breast cancer was examined by Kaplan-Meier plotter (http://kmplot.com/analysis).

### Statistical analysis

Statistical analyses were conducted using GraphPad Prism 8.0 software. Group comparisons were performed using appropriate statistical tests, such as Student’s t-test, one-way ANOVA, or two-way ANOVA. The correlation between ANGPTL4 mRNA levels and KLF4 density was assessed using Spearman’s correlation analysis. Data are expressed as mean ± standard error of the mean (S.E.M.) from at least three independent experiments, with a p-value < 0.05 considered statistically significant. For in vivo studies, “n” denotes the number of animals, as detailed in the figure legends.

## Results

### Adipocytes promote TNBC cells glycolysis, migration and invasion

**Fig. 1:**
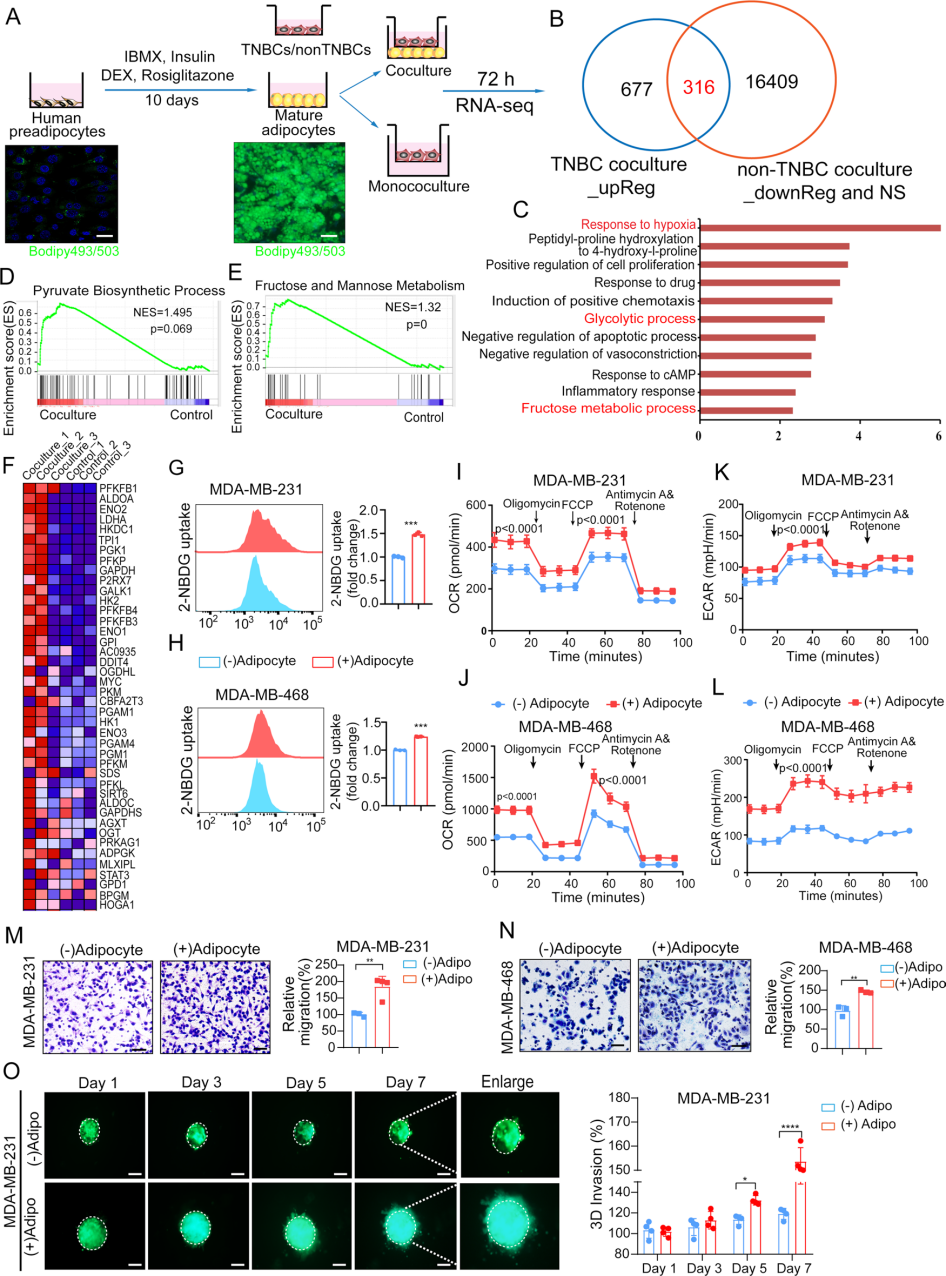
Adipocytes promote TNBC cells glycolysis, migration and invasion. (**A**) Schematic diagram of the human pre-adipocytes differentiation into mature adipocytes model and establishment of adipocytes-breast cancer co-culture system. (**B**) A Venn diagrams of differential expression genes in RNA sequencing of TNBC-coculture vs. TNBC(MDA-MB-468 cells) alone and non-TNBC-coculture vs. non-TNBC(BT474 cells) alone. (**C**) A KEGG bioinformatics analysis of RNA-seq data showing potential alterations in enriched signaling pathways. (**D-E**) Enrichment of pyruvate biosynthesis process and fructose and mannose metabolism process in TNBC cells after cocultured with adipocytes. (**F**) Heatmap illustrates differential expression patterns of pyruvate biosynthesis process in MDA-MB-468 cells after cocultured with human adipocytes for 72 h. (**G-H**) Glucose uptake was assessed using the 2-NBDG assay in TNBC cells, both with and without coculturing with adipocytes. (**I-L**) MDA-MB-231, MDA-MB-468 cells were co-cultured with mature adipocytes for 72 h, followed by measurement of OCR and ECAR, using Seahorse XFe96 analyzer. (**M-N**) MDA-MB-231 or MDA-MB-468 cells were either cocultured with adipocytes or monocultured for 72 h, after which the cancer cells were subjected to migration assays by transwell assay. Scale bar 100 μm. (O) MDA-MB-231 cells were cocultured with adipocytes or monocultured for 72 h, after which they were utilized in hanging drop matrix invasion assay. *p < 0.05; **p < 0.01; ***p < 0.001; ***p < 0.0001

To perform a comprehensive analysis of the differences between TNBC (MDA-MB-468) or non-TNBC(BT-474) breast cancer cells upon coculture with adipocytes, we profiled the transcriptomes of TNBC and non-TNBC cells with or without cocultured with mature adipocytes for 72 h (Fig. 1A). There were 677 upregulated genes in TNBC cells in the presence of adipocytes, while 16,409 downregulated or unchanged genes in non-TNBC cells after cocultured with adipocytes. Among these genes, 316 characteristically differentially expressed genes in TNBC cells after coculture rather than non-TNBC cells (Fig. 1B). Next, we explored KEGG analysis revealed that adipocytes led to the activation of hypoxia response, glycolytic process and fructose metabolism in TNBC cells (Fig. 1C). Gene set enrichment analysis (GSEA) revealed that adipocytes resulted in the elevation of pyruvate biosynthesis, fructose and mannose metabolism in cocultured TNBC cells (Fig. 1D-E). Next, we identified the genes involved glycolysis were elevated in TNBC cells upon treated with adipocytes (Fig. 1F).

These results highlight the metabolic plasticity of TNBC cells in response to adipocyte stimulation. To further investigate the interactions between adipocytes and cancer cells, we conducted combined gene and metabolite over-representation analyses. Given that glucose is the primary energy substrate for cancer cells, we performed 2-NBDG assays, which showed significantly higher glucose consumption in TNBC cells upon coculture with adipocytes (Fig. 1G-H). ATP production, a key indicator of cellular energy status, was also elevated in cocultured TNBC cells compared to monoculture controls (Supplementary Fig. 1A-B). ATP production is fueled by both mitochondrial oxidative phosphorylation (OXPHOS) and glycolysis through glucose consumption. In line with previous findings, Seahorse metabolic analysis revealed that TNBC cells cocultured with adipocytes exhibited significantly higher mitochondrial metabolism, as measured by oxygen consumption rate (OCR), and enhanced glycolysis, as indicated by extracellular acidification rate (ECAR), compared to monocultured cells. (Fig. 1I-L).

We subsequently assessed whether adipocytes could enhance TNBC cell proliferation and invasion through various assays. The EDU assay revealed that adipocytes increased the proliferation of MDA-MB-231 cells, but not MDA-MB-468 cells (Supplementary Fig. 1C-D). Transwell assays were subsequently employed to assess whether adipocytes enhanced the migration of TNBC cells. Co-culture of TNBC cells with adipocytes demonstrated a notable elevation in the migratory capacity of the cancer cells (Fig. 1M-N). In addition, when GFP-labeled TNBC cell spheroids were seeded onto a Matrigel matrix, adipocyte-exposed MDA-MB-231 cells exhibited significantly increased invasive potential (Fig. 1O). Collectively, these findings indicate that adipocytes promote glycolysis, migration, and invasion in TNBC cells.

### Adipocytes reprogram TNBC cell metabolism through activating ANGPTL4

**Fig. 2:**
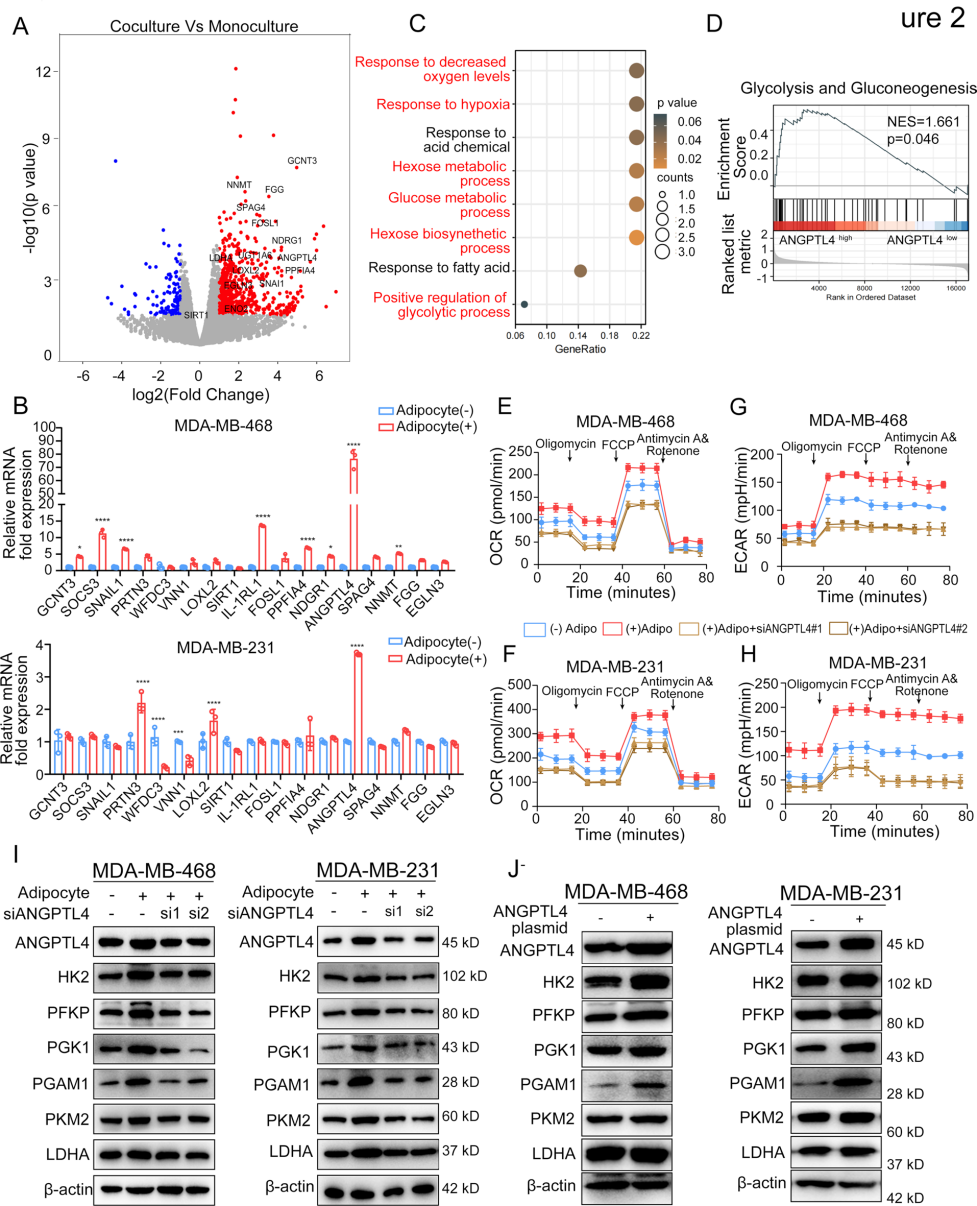
Adipocytes reprogram TNBC cell metabolism through regulating ANGPTL4. (**A**) Differential gene expression analysis was conducted between the TNBC coculture and monoculture groups. (**B**) qPCR assay was employed to assess the expression levels of the most significantly upregulated and downregulated genes in MDA-MB-231 and MDA-MB-468 cells, either alone or coculture with adipocytes. (**C**) Gene Set Enrichment Analysis based on the ANGPTL4 expression using the TCGA database. (**D**) ANGPTL4 was significantly enriched during glycolysis and gluconeogenesis process. (**E-H**) TNBC cells with ANGPTL4 knockdown were co-cultured with mature adipocytes for 3 days, followed by measurement of OCR and ECAR, using Seahorse XFe96 analyzer. (**I**) TNBC cells with ANGPTL4 knockdown were co-cultured with mature adipocytes to detect the protein expression of HK2, PFKP, PGK1, PKM2 and other glycolytic enzymes by Western Blot analysis. (**J**) The expression of HK2, PFKP, PGK1, LDHA, PKM2 and other glycolytic enzymes were detected in MDA-MB-231 or MDA-MB-468 cells after ANGPTL4 overexpression. *p < 0.05; **p < 0.01; ***p < 0.001

We further investigated how adipocytes specifically reprogram TNBC cell metabolism. Differential gene expression analysis revealed 1,311 upregulated genes and 574 downregulated genes in cocultured TNBC cells compared to monocultured TNBC cells (Fig. 2A). To validate these RNA-seq results, we measured the mRNA levels of the top 20 candidate genes in TNBC cells after 72 h of coculture with adipocytes. ANGPTL4 was identified as a key candidate due to its highest expression in TNBC cells upon coculture with adipocytes (Fig. 2B), interestingly, ANGPTL4 expression was also slightly elevated in non-TNBC cells in the presence of adipocytes (Supplementary Fig. 2A).

ANGPTL4 is widely recognized for its role in regulating lipid metabolism and maintaining glucose homeostasis [29]. To explore the impact of ANGPTL4 on breast cancer metabolism, KEGG analysis of breast cancer data from TCGA database revealed that ANGPTL4 expression is strongly associated with pathways involved in hypoxia, glucose metabolism, hexose metabolism, and glycolysis (Fig. 2C). Furthermore, we stratified bulk breast cancer data from TCGA into low and high ANGPTL4 expression groups and conducted GSEA analysis. A hallmark analysis unveil that the levels of glycolysis and gluconeogenesis gene sets was enriched in the high ANGPTL4 expression group (Fig. 2D). This led us to hypothesize that ANGPTL4 may mediate the metabolic plasticity of adipocyte-enriched TNBC cells. To test this hypothesis, we first examined whether ANGPTL4 expression correlates with mitochondrial metabolism and glycolytic activity. Plate-based respirometry analysis of oxygen consumption rates in TNBC cells with ANGPTL4 silencing in the presence of adipocytes, and found that ANGPTL4 knockdown in TNBC cells co-cultured with adipocytes led to a reduction of oxygen consumption (Fig. 2E-F) and glycolysis (Fig. 2G-H). Consistent with these findings, adipocytes increased both mRNA and protein levels of glycolytic enzymes, including hexokinase 2 (HK2), phosphofructokinase platelet (PFKP), phosphoglycerate kinase (PGK1), phosphoglycerate mutase 1 (PGAM1), and pyruvate kinase isozyme 2 (PKM2), in cocultured TNBC cells compared to monocultured cells (Supplementary Fig. 2B and Fig. 2I). However, ANGPTL4 knockdown reversed the effects of adipocytes on glycolytic enzyme expression (Fig. 2I). Conversely, ANGPTL4 overexpression in TNBC cells led to increased expression of HK2, PGK1 and PGAM1 (Fig. 2J). These results suggest that adipocyte coculture enhances glucose metabolism, with ANGPTL4 playing a key role in supporting metabolic reprogramming in TNBC cells.

### ANGPTL4 Blockade inhibits TNBC metastasis induced by adipocytes

**Fig. 3:**
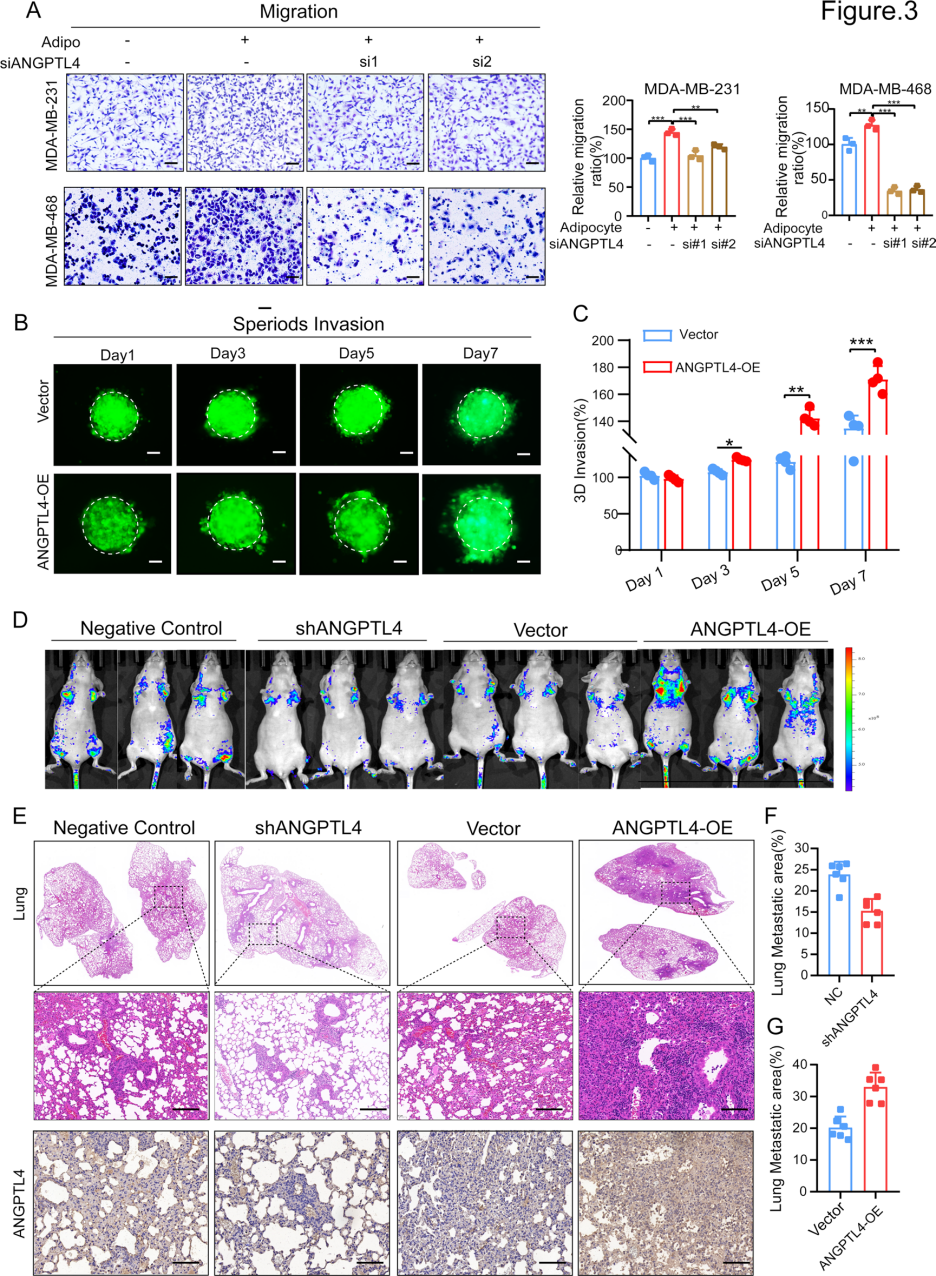
ANGPTL4 blockade inhibits TNBC metastasis induced by adipocytes. (**A**) Transwell assay to measure the contribution of adipocytes on migration of TNBC cells and the role of ANGPTL4 in this process. Scale bar 100 μm. (**B-C**) Hanging drop to assess ANGPTL4 overexpression on TNBC cells invasion with the indicated condition. Two-way ANOVA was used to analyze the invasion rate. (**D**) shRNA targeting human ANGPTL4 (shANGPTL4), with scramble shRNA-transfected cells serving as the negative control, and ectopic ANGPTL4 overexpression in MDA-MB-231 cells were tail intravenous injected into NOD/SCID mice and tumor burden visualized after 3 weeks using the IVIS spectrum in vivo imaging system. (**E-G**) Representative images of lungs detected by **H**&**E** staining. The number of lung metastases was calculated and ANGPTL4 expression in lung tissues was detected by immunohistochemistry staining. scale bars, 100 μm.Each group is *n* = 6. *p < 0.05; **p < 0.01; ***p < 0.001

To reveal the effect of ANGPTL4 knockdown on adipocyte-driven TNBC migration. We performed transwell assay, which demonstrated that ANGPTL4 silencing via siRNAs significantly impaired adipocyte-induced TNBC cell migration (Fig. 3A). In contrast, ectopic ANGPTL4 expression in TNBC cells showed a markedly increasing in migration of TNBC cells (Supplementary Fig. 3). To investigate ANGPTL4 on TNBC invasion, we plated GFP-labeled TNBC cells spheroids with ANGPTL4 overexpression seeded onto the Matrigel matrix surface, and it was showed that ectopic ANGPTL4 expression resulted in increased TNBC invasion within Matrigel at day 3, day 5 and day 7, with the pro-invasive effect of ANGPTL4 becoming more pronounced with prolonged invasion time (Fig. 3B-C).

To investigate the role of ANGPTL4 in TNBC metastasis in vivo, ANGPTL4-knockdown and ectopic ANGPTL4 expression breast cancer cells were intravenously injected via the tail vein into mice. Bioluminescence imaging revealed that ANGPTL4 knockdown significantly reduced pulmonary metastasis compared to the negative control group, while ectopic ANGPTL4 expression accelerated lung metastasis of breast cancer (Fig. 3D). Additionally, ANGPTL4 silencing decreased the number and size of metastatic lesions as observed in H&E staining sections (Fig. 3E-F). In contrast, ANGPTL4 overexpression resulted in larger and more numerous metastatic lesions in lung sections (Fig. 3E and G), with larger lesions exhibiting higher ANGPTL4 expression relative to smaller lesions (Fig. 3E). Collectively, these findings illuminate that ANGPTL4 plays a considerable role in adipocyte-driven metastasis of TNBC cells.

### Adipocyte-derived lipids activate PPARα/ANGPTL4 axis in TNBC cells

**Fig. 4:**
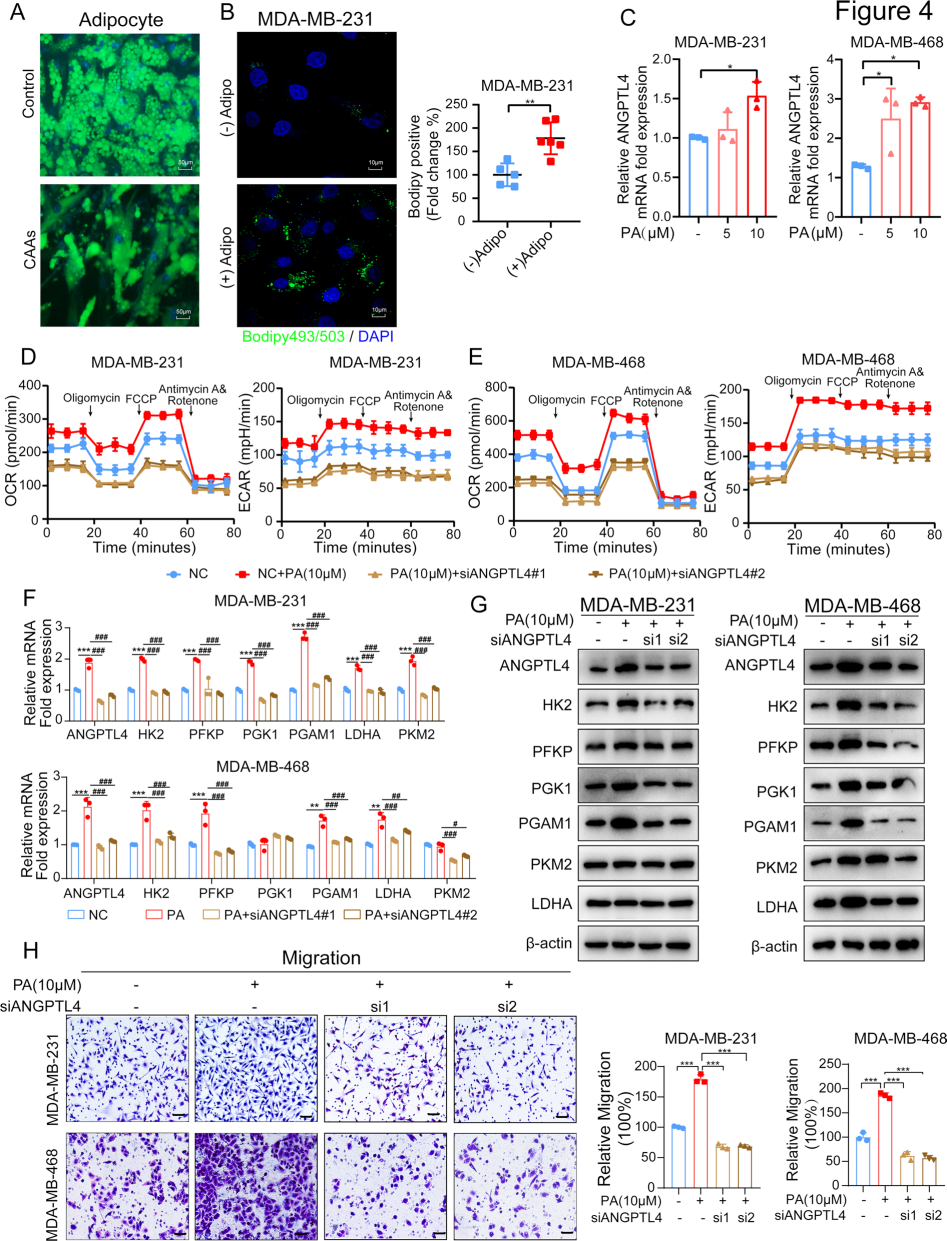
Adipocyte-derived lipids activate ANGPTL4 in TNBC cells. (**A-B**) Bodipy493/503 to measure the lipid in adipocytes and TNBC breast cancer cells. Representative immunofluorescent images of cellular lipid accumulation (lipid in green, BODIPY 493/503 and nucleus in blue, DAPI) are shown. (**C**) qPCR to assess ANGPTL4 gene expression in TNBC cells treatment with PA (5µM, 10µM) for 72 h. (**D-E**) siRNAs targeting ANGPTL4 (siANGPTL4#1, #2) were incubated with or without PA (10µM) in MDA-MB-231, MDA-MB-468 cells for 72 h. OCR and ECAR rate were measured with the Seahorse XFe96 Analyzer. Data are means ± s.e.m. of six replicates. (**F-G**) qPCR and Western blot analysis of glycolytic-related enzymes levels in MDA-MB-231, MDA-MB-468 cells with ANGPTL4 knockdown in the absence and presence of PA stimulation. (H) Transwell assay to assess the role of PA treatment on the migration ability of TNBC cells and ANGPTL4 blockade abolished the promotive effect induced by PA. Scale bar 100 μm. *p < 0.05; **p < 0.01; ***p < 0.001

ANGPTL4 expression is known to be regulated by various nutritional and metabolic conditions, including fasting and hypoxia [29, 30]. To investigate whether lipid-enriched adipocytes play a role in activating ANGPTL4 in TNBC cells, we assessed the bidirectional lipid transfer between breast tumors and surrounding adipocytes. Using the lipophilic fluorescent dye Bodipy 493/503, we observed a decline in both the abundance and size of lipid droplets in CAAs due to co-cultured with TNBC cells, consistent with previous findings showing adipocyte morphological changed into a “spindled” shape and dedifferentiation (Fig. 4A). In contrast, co-cultured MDA-MB-231 cells exhibited numerous small lipid droplets, as indicated by Bodipy 493/503 staining of neutral lipids (Fig. 4B). Lipid transfer from adipocytes to tumor cells occurs via FATPs, CD36, and FABP4, as previously reported [13, 24]. In our study, CD36 protein expression was significantly elevated in co-cultured TNBC cells (Supplementary Fig. 4), suggesting that fatty acids released by adipocytes may be transferred to TNBC cells, driving ANGPTL4-dependent metabolic reprogramming and migration.

Previous studies have demonstrated that palmitic acid (PA), oleic acid, and linoleic acid are the most abundant fatty acids residing in the adipocyte medium [31]. We treated TNBC cells with PA in vitro and found that PA treatment significantly upregulated ANGPTL4 mRNA levels, as confirmed by qRT-PCR (Fig. 4C). To further investigate the role of free fatty acids (FFAs) in TNBC cell metabolism, we utilized palmitic acid to stimulate TNBC cells with ANGPTL4 knockdown and performed Seahorse metabolic analysis. The results revealed that PA treatment enhanced both mitochondrial metabolism and glycolysis, whereas ANGPTL4 knockdown significantly impaired these metabolic processes in response to PA stimulation (Fig. 4D-E). Parallel to these observations, qRT-PCR and Western blotting demonstrated that PA treatment increased the expression of glycolytic enzymes, including HK2, PFKP, PGK1, and PGAM1, at both the transcriptional and protein levels (Fig. 4F-G). However, ANGPTL4 knockdown reversed the PA-induced upregulation of glycolytic enzymes (Fig. 4F-G). Additionally, transwell assays measuring TNBC cell migration revealed that ANGPTL4 knockdown significantly inhibited PA-mediated cell migration (Fig. 4H).

**Fig. 5:**
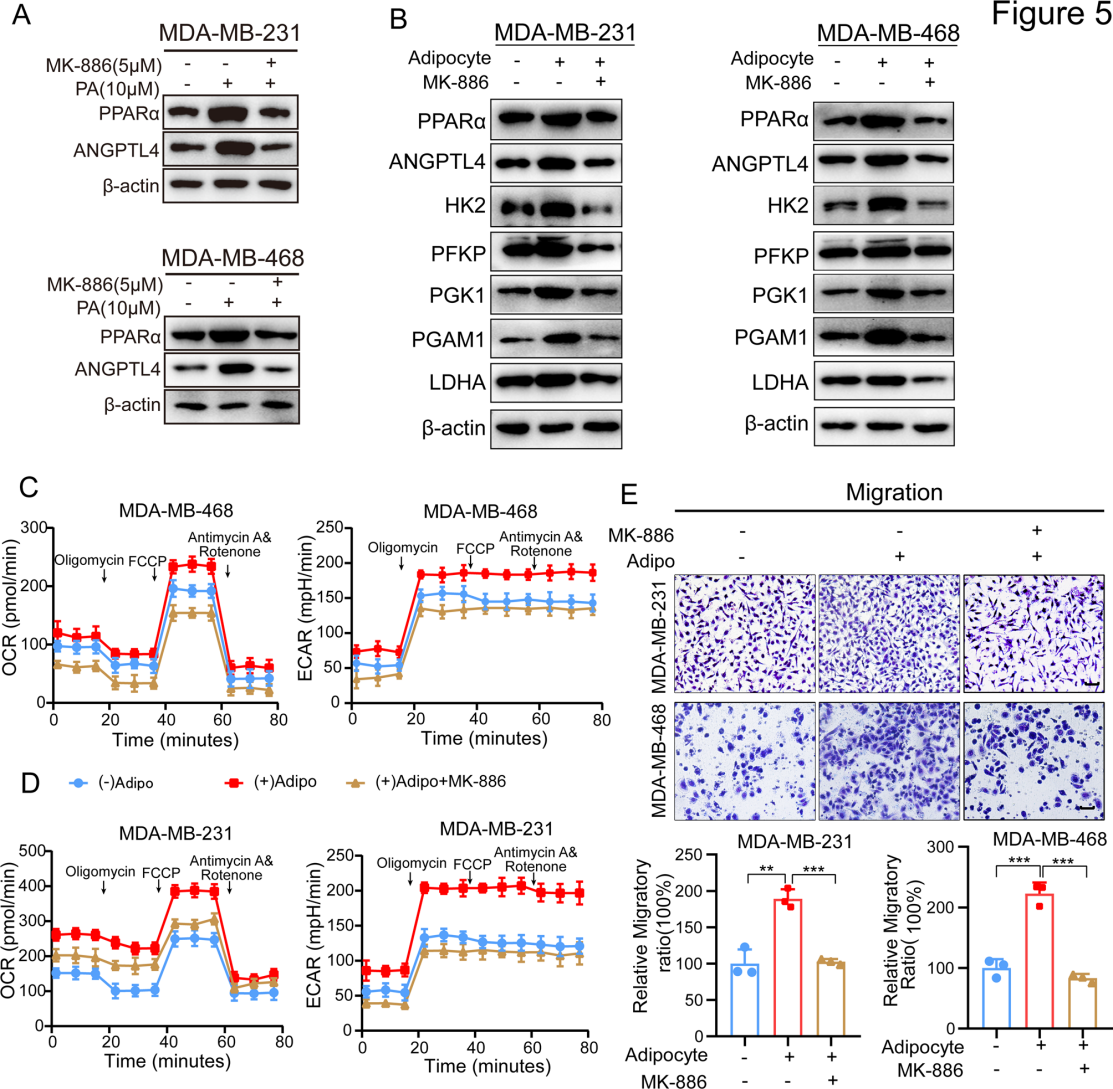
PPARα favors lipids-driven ANGPTL4 activation in TNBC cells. (**A**) Western blot analysis of PPARα and ANGPTL4 protein expression in TNBC cell lines stimulated with PA and or treated with the PPARα inhibitor MK-886 (5µM). (**B**) Western blot analysis of PPARα, ANGPTL4, HK2, PFKP, PGK1, PGAM1, LDHA in TNBC cells cocultured with adipocytes, or treated with MK-886 (5µM) in a co-culture system. (**C-D**) The OCR and ECAR levels of TNBC cells in each group were measured in TNBC cells cultured alone, co-cultured with adipocytes, or treatment of MK-886(5µM) in the co-culture system. (**E**) Transwell assay to measure the role of MK-886 treatment on the migration ability of TNBC cells induced by adipocytes. Scale bar 100 μm. *p < 0.05; **p < 0.01; ***p < 0.001

FFAs induce the activation of peroxisome proliferator-activated receptors (PPARs) [32], and we hypothesized that lipids produced by adipocyte lipolysis might activate PPARα, thereby regulating ANGPTL4 expression in TNBC cells. To test this hypothesis, we employed the PPARα inhibitor, MK886, to block the influence of PA on ANGPTL4 expression. Exposure of TNBC cells to PA induced the upregulation of both PPARα and ANGPTL4 protein expression, whereas MK886 effectively blocked PA-induced ANGPTL4 protein expression (Fig. 5A). To further investigate the function of PPARα in adipocyte-induced metabolic reprogramming, we examined its effect on ANGPTL4 and downstream glycolytic metabolism in co-cultured TNBC cells. PPARα inhibition led to a mitigation of both ANGPTL4 and glycolytic enzyme expression (Fig. 5B). Consistent with these findings, PPARα blockade also decreased adipocyte-induced mitochondrial oxygen consumption and glycolysis (Fig. 5C-D). Furthermore, transwell assays revealed that blocking PPARα significantly reversed adipocyte-driven TNBC cell migration (Fig. 5E). Collectively, these results suggest that lipids released by adipocytes transfer to TNBC cells and regulate their metabolic phenotype via the activation of the PPARα/ANGPTL4 axis.

### Adipocytes-derived IL6 drives STAT3/ANGPTL4 pathway in TNBC cells

**Fig. 6:**
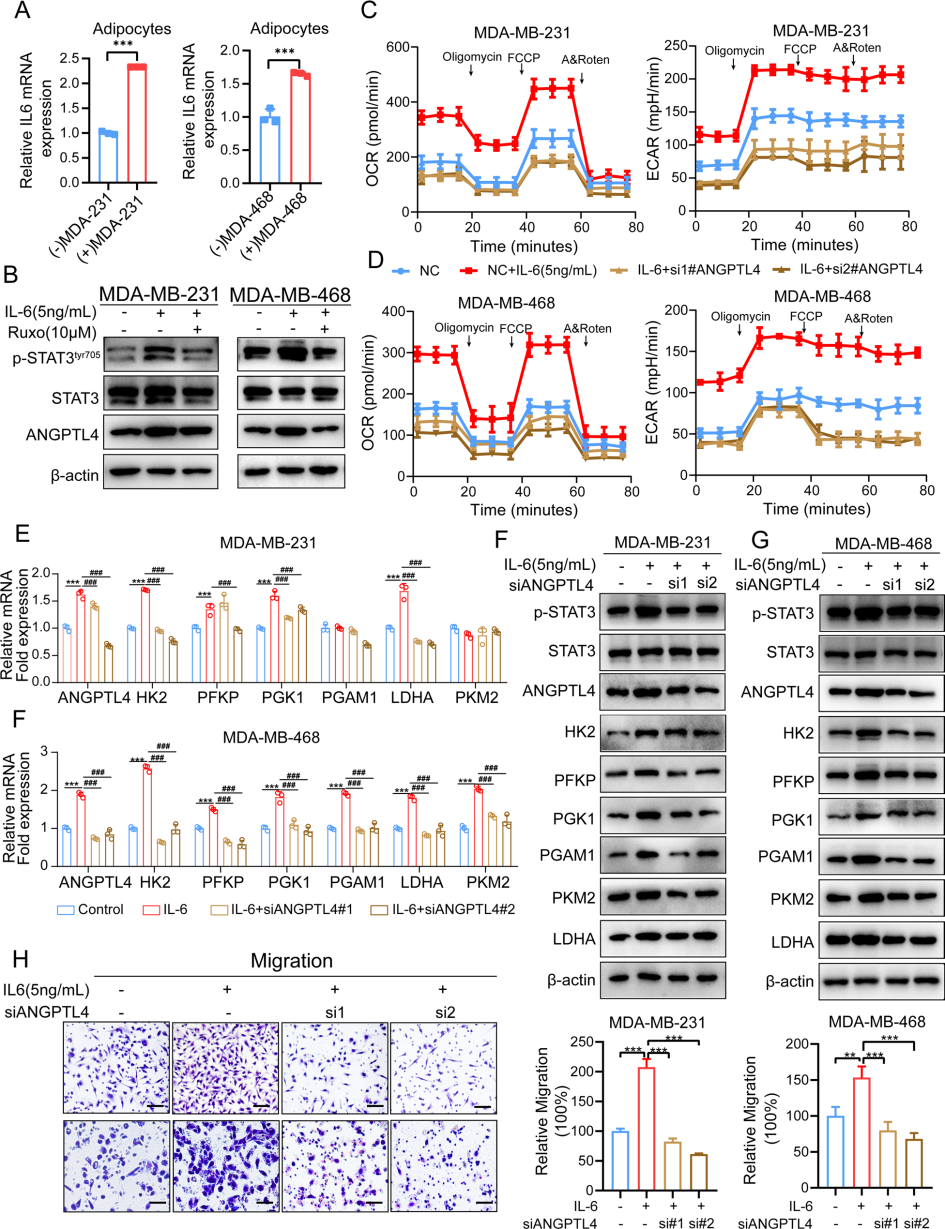
Adipocytes derived IL6 drives STAT3/ANGPTL4 pathway in TNBC cells (**A**) qPCR analysis to assess IL6 gene expression in adipocytes cocultured with breast cancer cells or monoculture. (**B**) Western blot analysis of phosphorylation STAT3 at Tyr705, total STAT3 and ANGPTL4 protein expression in TNBC cell lines stimulated with human recombinant IL6 or treated with the JAK inhibitor Ruxo (5µM). (**C-D**) The OCR and ECAR levels were measured in TNBC cells cultured alone, stimulating with IL-6 (5ng/mL), or ANGPTL4 knockdown cells with IL-6 (5ng/mL) stimulation. (**E-F**) qPCR analysis to measure mRNA levels of ANGPTL4 and glycolytic-related enzymes in TNBC cells with ANGPTL4 knockdown, following stimulation with IL-6 (5 ng/mL) for 3 days. (**G-H**) Western blot analysis protein expression of phosphorylation STAT3 at Tyr705, total STAT3, ANGPTL4 and glycolytic-related enzymes in TNBC cells with ANGPTL4 knockdown, following stimulation with IL-6 (5 ng/mL) for 3 days. (**I**) Transwell assay to measure the role of ANGPTL4 on IL6-induced TNBC cells migration. Scale bar 100 μm. *p < 0.05; **p < 0.01; ***p < 0.001

We and others have demonstrated that adipocytes secrete various cytokines and adipokines that contribute to adipose-enriched cancer progression [17]. Interleukin-6 (IL-6), a prominent cytokine in the tumor microenvironment (TME) of breast cancer (TNBC), has been implicated in promoting TNBC metastasis [33]. Previous studies have shown that adipocyte-derived IL-6 activates hypoxia-inducible factor 1-alpha (HIF1α) in ovarian cancer, leading to increased tumor cell glycolysis [28]. Concordant with these findings, we noticed that in co-cultured adipocytes, IL-6 mRNA expression was significantly elevated compared to adipocytes cultured alone (Fig. 6A), and our previous study showed adipocytes or cancer-associated adipocytes (CAAs) secreted IL-6 in the adipocyte-conditioned medium [33], suggesting that the crosstalk between TNBC cells and adipocytes enhances IL-6 expression in cancer-associated adipocytes (CAAs). To explore the role of IL-6 and its downstream STAT3 signaling in regulating ANGPTL4 expression in TNBC cells, we treated TNBC cells with human recombinant IL-6 and found an upregulation protein expression of ANGPTL4 and STAT3 phosphorylation (Fig. 6B). Inhibition of IL-6 signaling using a JAK kinase inhibitor resulted in a reduction of IL-6-induced ANGPTL4 expression (Fig. 6B). To investigate whether the IL-6 pathway contributes to TNBC cell glycolysis, we performed Seahorse metabolic analysis, which revealed that recombinant IL-6 treatment significantly enhanced both mitochondrial OXPHOS and glycolysis in TNBC cells. However, this effect was abrogated by ANGPTL4 knockdown (Fig. 6C-D). Consistent with these findings, IL-6 stimulation upregulated the mRNA and protein levels of glycolytic enzymes, including hexokinase 2 (HK2), phosphofructokinase platelet (PFKP), phosphoglycerate kinase 1 (PGK1), and pyruvate kinase isozyme M2 (PKM2), while ANGPTL4 knockdown significantly reduced the expression of these glycolytic enzymes (Fig. 6E-G).

To further elucidate whether IL-6-driven migration of TNBC cells is partially dependent on ANGPTL4 expression, we stimulated TNBC cells with human recombinant IL-6 and observed that IL-6 significantly enhanced TNBC cell migration. However, ANGPTL4 knockdown markedly attenuated IL-6-induced migratory capacity (Fig. 6H). Collectively, these results suggest that adipocytes may promote ANGPTL4 expression in TNBC cells via an IL-6-mediated JAK/STAT3 signaling pathway.

### ANGPTL4 regulates transcriptional factor KLF4 to favor Glycolysis and metastasis

**Fig. 7:**
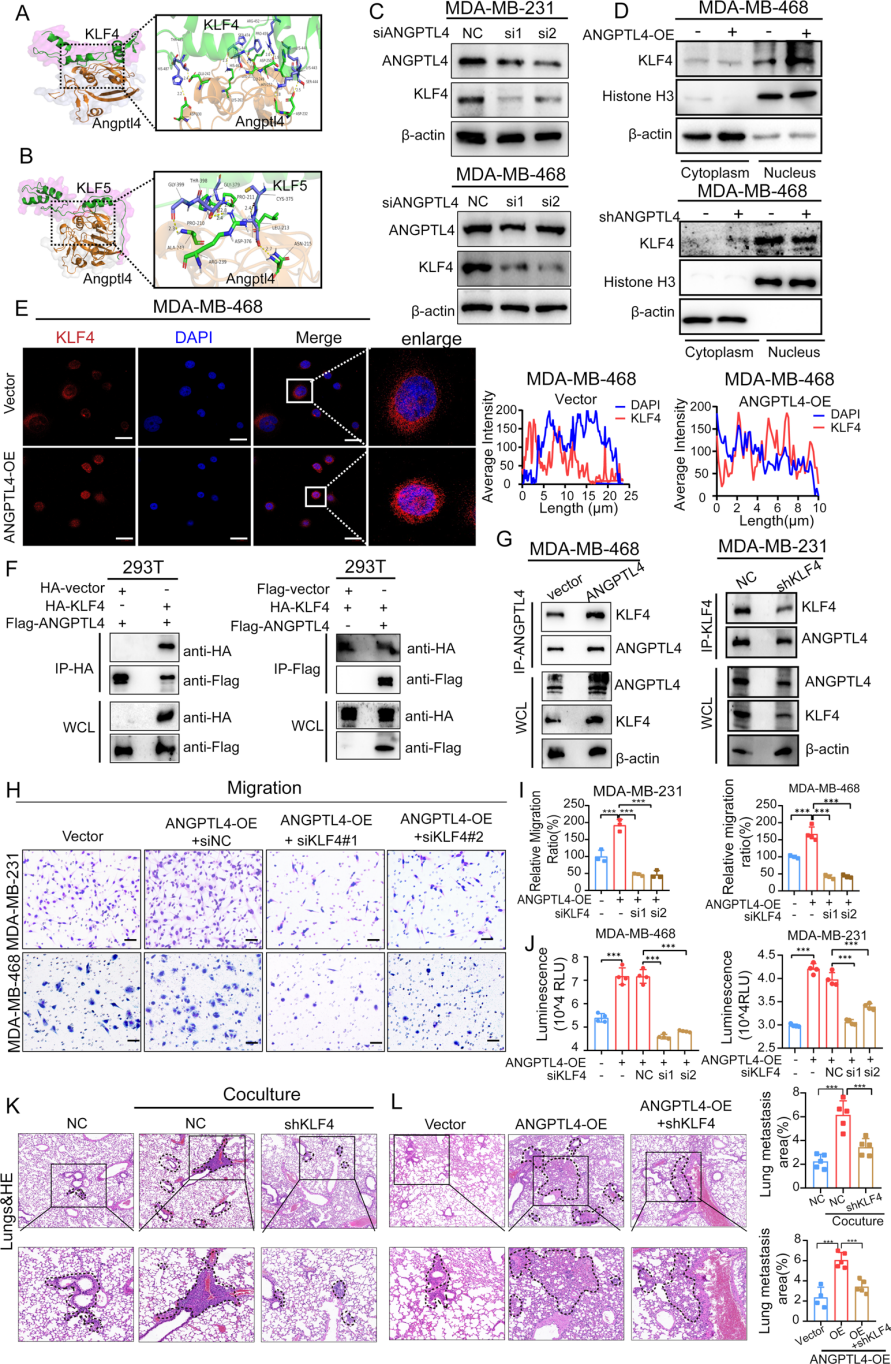
ANGPTL4 regulates transcriptional factor KLF4 to favor glycolysis and metastasis. (**A-B**) Visualization of protein-protein docking results of transcription factors KLF4 and KLF5 with ANGPTL4, respectively. (**C**) Western blot analysis of ANGPTL4 and KLF4 in TNBC cell lines with ANGPTL4 knockdown. (**D**) The nuclear translocation KLF4 through the separation of the cytoplasm and nuclear proteins of MDA-MB-468 cells with ANGPTL4 overexpression or knockdown. (**E**) Immunofluorescent assay to detect subcellular distribution of KLF4 in TNBC cells (Vector) and ANGPTL4-overexpressing cells (ANGPTL4-OE). Nucleus stained with DAPI (blue). Scale, 20 μm. (**F**) Co-IP assay showing the exogenous interaction between ANGPTL4 and KLF4 in 293T cells transfected with Flag-ANGPTL4 or HA-KLF4. (**G**) Co-IP assay showing the endogenous interaction between ANGPTL4 and KLF4 in ANGPTL4 overexpression MDA-MB-468 cells, or KLF4-knockdown MDA-MB-231 cells. (**H-I**) Transwell assay was performed to evaluate the role of KLF4 on ANGPTL4-driven TNBC cells migration. (**J**) Total ATP levels were detected in TNBC cells with ANGPTL4 overexpression accompanied with or without KLF4 knockdown. (**K**) MDA-MB-231 cells were monocultured, cocultured with adipocytes, or cocultured with adipocytes following KLF4 depletion. After 72 h, these cells were harvested, and resuspended in serum free media to a concentration of 5 × 10^5 cells per 200µL, and injected into tail vein of each Balb/c mouse. After 14 days, the mice were sacrificed and lungs were isolated and sectioned for histological studies. Hematoxylin and eosin (H&E) staining of transverse sections of lungs from mice injected with MDA-MB-231 cells monocultured, cocultured with adipocytes, cocultured with adipocytes, or cocultured with adipocytes following KLF4 depletion. Scale bar 100 μm. *p < 0.05; **p < 0.01; ***p < 0.001

Considering that ANGPTL4 regulates glycolytic enzymes at both the transcriptional and protein levels, we hypothesized that the function of ANGPTL4 may be mediated by transcription factors. Kruppel-like transcription factors (KLFs), which play a key role in maintaining pluripotency [34], and KLF4/KLF5 have been shown to be elevated in aggressive primary breast tumors [35, 36], promoting metabolic reprogramming and tumor progression [8, 37, 38]. Moreover, in smooth muscle cells (SMCs), ANGPTL4 could inhibit KLF4 expression to block SMCs differentiation into macrophage-like cells [39]. However, the regulation role of ANGPTL4 on KLF4/KLF5 in TNBC cells has not been studied. To evaluate the potential interaction between KLF4/KLF5 and ANGPTL4, we performed molecular docking analysis between ANGPTL4 and KLF4/KLF5. Computational modeling revealed that residues Asp330, Glu242, Lys261, Leu213, Asp250, His252, Gly233, and Asp231 in ANGPTL4 interact with His487, Thr485, Ser474, His461, Pro459, Arg452, Lys448, and Lys443 in KLF4 through hydrogen bonds (Fig. 7A), with an E_dock value of -8.938. Similarly, residues Ala243, Pro210, Pro211, Leu213, and Asn215 in ANGPTL4 form hydrogen bond interactions with Gly399, Thr398, Gly379, Cys375, and Asp376 in KLF5, with an E_dock value of -23.268 (Fig. 7B). These findings suggest that the binding affinity between ANGPTL4 and KLF4 is stronger than that between ANGPTL4 and KLF5, as indicated by the respective docking scores (Fig. 7A-B).

To further investigate the regulatory role of ANGPTL4 on KLF4 and KLF5, we analyzed the mRNA and protein expression profiles of KLF4 and KLF5 in TNBC cells. Notably, ectopic ANGPTL4 expression led to a notable increase in KLF4 (approximately 4folds) and KLF5 (approximately 2folds) mRNA expression (Supplementary Fig. 5A). Conversely, ANGPTL4 knockdown resulted in a decrease in KLF4 expression, while KLF5 expression remained unaffected (Supplementary Fig. 5B). These findings were corroborated at the protein level, where KLF4 protein expression was notably decreased following ANGPTL4 knockdown in TNBC cells (Fig. 7C). Additionally, KLF4 expression was elevated in TNBC cells co-cultured with adipocytes, both at the mRNA and protein levels (Supplementary Fig. 5C, D). As transcription factors function through nuclear translocation, we subsequently assessed the influence of ANGPTL4 on the subcellular distribution of KLF4 in TNBC cells. As shown in Fig. 7D, we measured the protein levels of KLF4 in nuclear and cytosolic fractions in MDA-MB-468 TNBC cells, ANGPTL4 overexpression promoted KLF4 nuclear translocation, whereas ANGPTL4 knockdown inhibited the nuclear accumulation of KLF4 in TNBC cells. Similar results were obtained by immunofluorescence staining, the nuclear accumulation of KLF4 was markedly enhanced upon ANGPTL4 overexpression in TNBC cells compared to its counterparts (Fig. 7E).

To elucidate the molecular mechanisms underlying ANGPTL4-mediated KLF4 activation, co-immunoprecipitation assays were conducted in 293T cells transfected with Flag-tagged ANGPTL4 and HA-tagged KLF4. Reciprocal immunoprecipitation confirmed the interaction between ANGPTL4 and KLF4 (Fig. 7F). To validate this interaction in TNBC cells, co-immunoprecipitation was performed in MDA-MB-468 cells transfected with ANGPTL4 overexpression plasmid. As shown in Fig. 7G, ANGPTL4 overexpression enhanced the endogenous interaction with KLF4. Conversely, KLF4 knockdown inTNBC cells attenuated this interaction between KLF4 and ANGPTL4. Collectively, these findings suggest a reciprocal regulatory loop between ANGPTL4 and KLF4 in TNBC cells.

To further determine whether KLF4 contributes to the metabolic reprogramming induced by ANGPTL4, we assessed ATP production in TNBC cells. Initially, we observed a reduction in ATP levels following ANGPTL4 knockdown in TNBC cells (Supplementary Fig. 5E). In contrast, ectopic overexpression of ANGPTL4 resulted in increased ATP production, however, this ANGPTL4-driven enhancement of ATP production was abolished by KLF4 knockdown (Fig. 7J). Additionally, we examined whether KLF4 plays a role in enhancing glycolysis driven by ANGPTL4 in TNBC cells. Key glycolytic enzymes, such as HK2, were analyzed in TNBC cells with ectopic ANGPTL4 expression. The results elucidated that ANGPTL4 overexpression enhanced the protein levels of HK2, whereas KLF4 knockdown reduced the expression of these proteins in ANGPTL4-overexpressing cells, suggesting that KLF4 may be involved in the glycolytic regulatory effects of ANGPTL4 (Supplementary Fig. 5F-G).

Besides, to determine whether KLF4 mediates the pro-migratory effects of ANGPTL4, migration assays were performed in TNBC cells. As shown in Fig. 7H–I, KLF4 silencing significantly reduced cell migration compared to ANGPTL4 overexpression alone. To further assess the role of KLF4 in adipocyte-induced metastasis, tail vein injection assays were conducted using MDA-MB-231 cells previously cocultured with or without adipocytes, or with adipocytes following KLF4 knockdown. After 72 h of coculture, cells were harvested and injected intravenously into BALB/c nude mice. As shown in Fig. 7K, the dissemination of TNBC cells toward the lungs were enhanced in mice injected with MDA-MB-231 cells previously cocultured with adipocytes compared with mice with MDA-MB-231 cells grown alone, whereas KLF4 depletion abrogated the pro-metastatic effect of adipocytes. Additionally, to evaluate whether KLF4 is required for ANGPTL4-induced metastasis, MDA-MB-231 cells with ANGPTL4 overexpression, with or without KLF4 knockdown, were subjected to tail vein injection. As shown in Fig. 7L, KLF4 depletion significantly impaired ANGPTL4-driven lung metastasis. Collectively, these results indicate that KLF4 is essential for both adipocyte- and ANGPTL4-mediated TNBC metastasis.

### The expression of ANGPTL4 serves as a potential prognostic marker in breast cancer patients

**Fig. 8:**
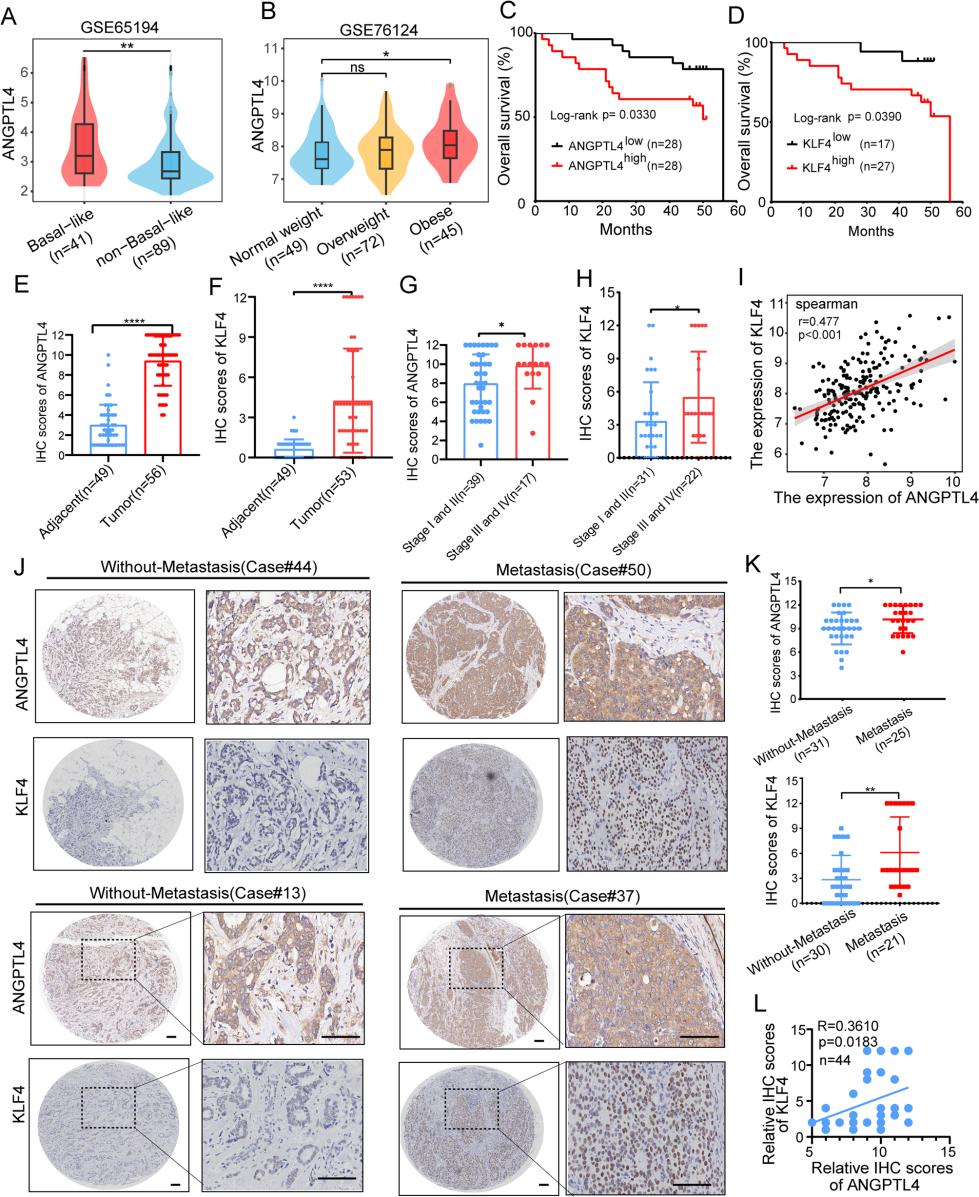
ANGPTL4 and KLF4 is associated with prognosis in patients with breast cancer. (**A**) ANGPTL4 expression was analyzed in the database (GSE65194) for basal-like (*n* = 41) and non-basal-like breast cancer (*n* = 89). (**B**) The ANGPTL4 expression in different BMI breast cancer was analyzed in the database (GSE76124), patients were divided into normal weight group (*n* = 49) (18 ≤ BMI < 25), overweight group (*n* = 72) (25 ≤ BMI < 30) and obese group (*n* = 45) (BMI ≥ 30) according to BMI size. (**C-D**) Survival analysis of ANGPTL4 and KLF4 expression on specimens’ overall survival. (**E**) The analysis of ANGPTL4 expression in breast cancer tissue(*n* = 56) and adjacent tissue(*n* = 49) was analyzed by immunohistochemistry (IHC). adjacent tissue refers to the tissue far than 2 cm away from the tumor foci. (**F**) The analysis of KLF4 expression in breast cancer tissue(*n* = 53) and adjacent tissue(*n* = 49) was analyzed by immunohistochemistry (IHC). adjacent tissue refers to the tissue far than 2 cm away from the tumor foci. (**G-H**) Scatter plots showing the staining index of ANGPTL4 and KLF4 in different stages status of breast cancer patients. (**I**) Correlation analysis of ANGPTL4 and KLF4 in breast cancer patients based on GSE76124. (**J-K**) IHC for ANGPTL4 and KLF4 expression in the non-metastatic and metastatic breast cancer tissues (Scale bars, 100 μm). (**L**) Correlation analysis of ANGPTL4 and KLF4 protein expression in the breast cancer tissues microarray (*n* = 44)

Our findings highlight the critical role of ANGPTL4 in driving tumor progression within the framework of adipocyte-accelerated breast cancer. To further assess whether ANGPTL4 and its downstream signaling pathways influence clinical outcomes in human breast cancer, we analyzed several annotated breast tumor datasets to compare ANGPTL4 expression across different breast cancer subtypes and among patients with varying body mass index (BMI). We observed that, irrespective of obesity status, ANGPTL4 expression was notably elevated in basal-like breast cancer compared to non-basal-like subtypes (Fig. 8A). Meaningfully, ANGPTL4 expression was also elevated in breast cancer tissues from obese patients (Fig. 8B). Moreover, Kaplan–Meier survival analysis of patient specimens revealed that high ANGPTL4 expression was associated with poorer overall survival, particularly in patients with basal-like breast cancer (Supplementary Fig. 6A-B).

Furthermore, we validated ANGPTL4 and KLF4 protein expression in breast cancer patients using immunohistochemistry (IHC) on a tissue microarray comprising of 58 breast cancer samples and 49 adjacent normal breast tissues. As anticipated, ANGPTL4 staining was predominantly observed in the cytoplasm and extracellular space of patient specimens, KLF4 staining was predominantly observed in the nucleus (Supplementary Fig. 6C). In survival analysis, breast cancer patients with elevated ANGPTL4 or KLF4 protein levels had poorer overall survival relative to those with low ANGPTL4 or KLF4 expression (Fig. 8C-D). It was showed that ANGPTL4 and KLF4 showed significantly higher expression levels in breast tumor tissues compared to adjacent normal tissues (Fig. 8E-F and Supplementary Fig. 6C). Additionally, ANGPTL4 and KLF4 expression was found to be significantly higher in patients at stage III-IV compared to those at stage I-II (Fig. 8G-H). Furthermore, correlation analysis unveiled a positive correlation between ANGPTL4 and KLF4 expression in breast cancer specimens (Fig. 8I). Moreover, patients with metastatic breast cancer exhibited elevated ANGPTL4 and KLF4 expression compared to those without metastasis (Fig. 8J-K). Specifically, ANGPTL4 and KLF4 were both highly expressed in metastatic breast cancer (Fig. 8J), and further co-expression analysis indicated that ANGPTL4 and KLF4 were simultaneously expressed in breast cancer tissue microarray (*R* = 0.3610, *p* = 0.0183, *n* = 44) (Fig. 8L). Notably, in metastatic breast cancer, adipocytes in proximity to breast cancer cells appeared smaller and contained fewer lipid droplets (Supplementary Fig. 6D). These findings indicate that ANGPTL4 serves as a negative prognostic factor in breast cancer, correlating with unfavorable clinicopathological parameters, including T stage and TNM stage.

## Discussion

**Fig. 9:**
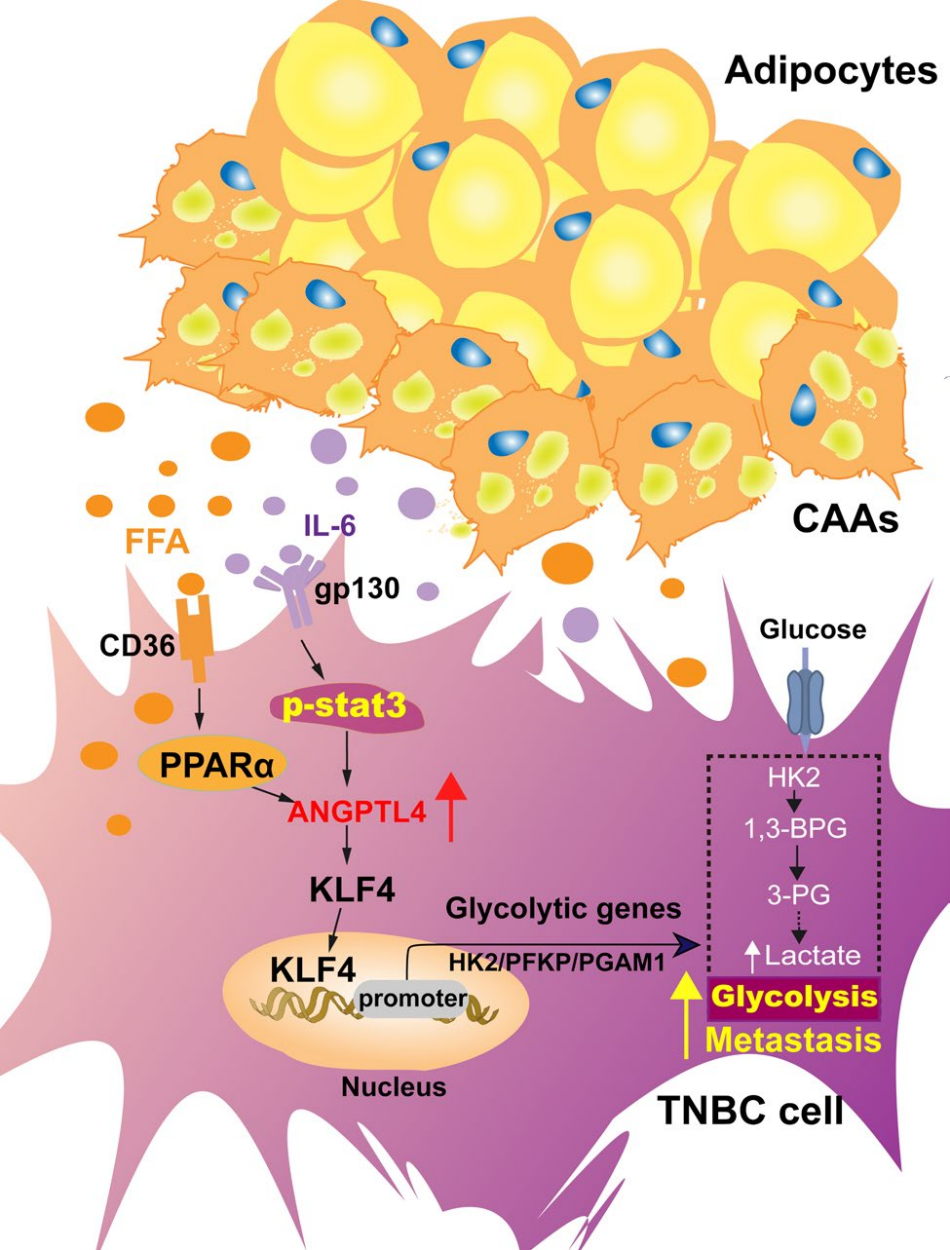
Molecular mechanism of the reciprocal between adipocyte and TNBC cells to favor glycolysis and metastasis

Recent studies have elucidated the pivotal function of the reciprocal between cancer cells and TME in driving the abnormal generation and transfer of metabolic intermediates, such as lactate and fatty acids, which cancer cells utilize for energy production [15, 19, 24]. Adipocytes play a critical role in supporting the elevated energy and biosynthetic demands of cancer cells through adipokine secretion and lipolysis [17, 19]. However, the underlying mechanisms by which adipocytes reprogram TNBC metabolism and promote metastasis remain poorly understood. In the current study, we demonstrate that ANGPTL4 function as a key regulator of metastasis and glycolysis, activated through interactions with adipocyte-enriched TNBC cells. Mechanistically, cancer-associated adipocytes (CAAs) secrete IL-6 and release free fatty acids (FFAs), which serve as metabolic sensors to activate ANGPTL4 via STAT3 and PPARα signaling pathways in TNBC cells. Furthermore, ANGPTL4 promotes the activation of the transcription factor KLF4, enhancing TNBC cell glycolysis and metastatic potential (Fig 9). This adipocyte-driven signaling cascade, involving the secretion of metabolites and cytokines, facilitates the invasion of TNBC cells. We propose that targeting ANGPTL4 represents a promising therapeutic strategy for inhibiting metastasis in TNBC.

Abundant studies have stressed the pivotal role of CAAs in advancing our awareness of the link between adipocytes and cancer progression, with adipocytes being central to the metabolic reprogramming of tumor cells [2, 19]. Specifically, adipocytes facilitate fatty acid oxidation (FAO), which contributes to the metabolic remodeling of tumor cells and promotes their progression [19, 22]. For instance, adipocytes serve as a lipid source, enhancing the expression of CD36, CPT1A/B, FATPs, and FABPs in tumor cells, thereby promoting FAO, which in turn supports tumor cell proliferation and invasive capabilities [13, 22, 23, 40]. In addition to inducing FAO, adipocytes also enhance glycolysis in breast and ovarian cancers [22, 28]. However, the underlying mechanisms through which adipocytes induce glycolysis in breast cancer remain poorly understood. In this study, we demonstrate that ANGPTL4 is likely responsible for mediating glycolysis in adipocyte-enriched TNBC cells, as ANGPTL4 knockdown reduced both glycolysis and metastasis in TNBC cells co-cultured with adipocytes. ANGPTL4 has been widely recognized as an oncogenic protein in both cancer and stromal cells [41–43]. Previous studies have shown that ANGPTL4 promotes metastasis upon breast cancer cell injection [43], supports energy production during epithelial-to-mesenchymal transition (EMT), and favors proliferation, migration, and invasion in non-small cell lung cancer (NSCLC) cells [44]. Furthermore, cancer-associated fibroblasts (CAFs) secrete ANGPTL4 as a mediator to promote breast cancer and prostate cancer progression [45, 46]. Additionally, as a regulator of lipid metabolism, ANGPTL4 reprograms lipid metabolism and promotes ovarian cancer metastasis [47]. Consistent with our findings, Zheng et al. identified ANGPTL4 as an upstream regulator of F. nucleatum-mediated glycolysis activation in colorectal cancer cells [48]. Moreover, adipose-derived stem cells (ADSCs) have been shown to elevate ANGPTL4 expression to mediate glycolysis in colorectal cancer cells [49].

It is noteworthy that ANGPTL4 expression is induced by fasting and hypoxia in a tissue-specific manner. Several transcription factors, including PPARs, glucocorticoid receptors, and HIF1α, have been identified as regulators of ANGPTL4 expression [29]. In the TME,cytokines such as TGFβ and IL-6 have been shown to modulate ANGPTL4 expression in cancer cells [42, 46]. Consistent with these findings, we demonstrate that IL-6 produced by CAAs plays a functional role in ANGPTL4 activation through the activation of the STAT3 signaling pathway in triple-negative breast cancer (TNBC) cells. Consistently, previous studies have established that STAT3 activation is responsible for ANGPTL4 expression in cancer-associated fibroblasts (CAFs) [45]. Moreover, recent research has shown that IL-6 secreted by adipocytes drives STAT3 signaling to induce HIF1α in cancer cells, with HIF1α known to regulate ANGPTL4 expression [28]. Interestingly, our results showed ANGPTL4 knockdown reduces STAT3 phosphorylation, suggesting a potential feedback loop between ANGPTL4 and STAT3 in TNBC cells. Li et al. reported that ANGPTL4 promotes ovarian cancer progression via activation of the JAK2/STAT3 pathway [47]. Similarly, in the colorectal cancer microenvironment, ANGPTL4 overexpression in endothelial cells activated JAK2/STAT3 signaling, enhancing angiogenesis and metastasis [50]. Beyond oncology, ANGPTL4 has also been implicated in STAT3 regulation in polycystic ovary syndrome and diabetes [51, 52], indicating its broader role in modulating JAK/STAT3 signaling across diseases. Beyond cytokine interactions, our findings also suggest that free fatty acids (FFAs) released by adipocytes contribute to the promotion of ANGPTL4 expression and glycolysis through PPARα signaling. These observations align with previous studies indicating that in the presence of FFAs, breast cancer cells adopt an energy-efficient phenotype, coping with metabolic stress by enhancing both their aerobic and glycolytic metabolic capacities. Notably, cells exhibiting this phenotype demonstrates increased glycolysis and augmented mitochondrial metabolism. Interestingly, PPARα has been identified as the primary PPAR subtype involved in the obesity-induced upregulation of ANGPTL4 expression in breast cancer cells [31]. Therefore, our results substantiate the role of FFAs provided by adipocytes in inducing ANGPTL4 expression in cancer cells by acting as ligands for PPARα. Additionally, it is noteworthy that PPARα has been reported to suppress HIF1α protein expression in cancer cells, despite HIF1α being critical for the regulation of glycolysis in living cells [53]. This suggests that HIF1α could be a particularly potent target for modulating ANGPTL4 expression and rewiring metabolism, as it is co-regulated by both IL-6-STAT3 and FFAs-PPARα signaling pathways.

Additionally, ANGPTL4 has been shown to activate FAK signaling in breast cancer cells treated with adipocyte-conditioned medium [31]. However, the mechanisms by which ANGPTL4 modulates glycolysis and metastasis in TNBC cells remain unclear. In this study, we identified KLF4 as a prospective downstream target of ANGPTL4, responsible for regulating glycolysis and migration in TNBC cells. KLF4 has been reported as a tumor suppressor in colon adenomas, gastrointestinal tumors, lung cancer [54]. Nonetheless, KLF4 is overexpressed in approximately 70% of primary breast ductal carcinomas, implicating its oncogenic role in breast cancer progression [55]. The increased nuclear expression of KLF4 has been associated with the aggressiveness of breast cancer phenotypes [35, 56]. Yu et al. demonstrated that KLF4 knockdown delayed tumor development and decreased pulmonary metastasis, while KLF4 expression levels served as a prognostic marker for stratifying HER2-enriched breast cancer according to distant metastasis-free survival [37, 57]. In breast cancer, KLF4 contributes to the maintenance of high glycolytic metabolism through the transcriptional activation of the PFKP gene, which further supports breast cancer progression [38]. In line with these findings, we currently reveal that KLF4 protein expression is elevated in TNBC cells co-cultured with adipocytes, alongside increased ANGPTL4 expression and glycolytic enzyme expression, such as HK2 and PFKP. Importantly, KLF4 knockdown abrogated ATP production and reduced the expression of HK2 and PFKP, which were otherwise induced by ANGPTL4 overexpression. These findings suggest that KLF4 may function as a downstream transcriptional factor of ANGPTL4, mediating the regulation of glycolysis. Interestingly, our results revealed that KLF4 also regulates ANGPTL4 expression. Specifically, KLF4 knockdown reduces ANGPTL4 expression and weakened the interaction between ANGPTL4 and KLF4. These findings suggest a potential positive feedback loop between ANGPTL4 and KLF4. However, the precise molecular mechanism linking ANGPTL4 and KLF4 remains to be fully elucidated, and further investigation is necessary to clarify the underlying regulatory pathway.

In conclusion, our findings elucidate that adipocyte contributes to the metabolic reprogramming and metastatic potential of TNBC cells through activating ANGPTL4. We demonstrate that adipocyte-derived interleukin-6 (IL6) and free fatty acids (FFAs) are crucial in triggering the STAT3 and PPARα signaling pathways, leading to the upregulation of ANGPTL4 in TNBC cells. Furthermore, we identify that ANGPTL4 interacts with the transcription factor KLF4, thereby driving the metabolic reprogramming and metastatic behavior of TNBC cells. Our analysis of clinical datasets reveals that ANGPTL4 and KLF4 expression are significantly elevated in TNBC patients, particularly those with obesity, and correlates with poor prognosis. These findings suggest that ANGPTL4 could act as a promising prognostic biomarker and represents a potential therapeutic target for the treatment of TNBC patients.

## Electronic supplementary material

Below is the link to the electronic supplementary material.

Supplementary Material 1

Supplementary Material 2

Supplementary Material 3

## Data Availability

Data is provided within the manuscript or supplementary information files.

## Abbreviations

TME: Tumor microenvironment;
CAAs: Cancer-associated adipocytes;
TNBC: Triple-negative breast cancer,
ANGPTL4: Angiopoietin-like 4;
IL6: Interleukin 6;
KLFs: Kruppel-like transcription factors;
PPARs: Peroxisome proliferator-activated receptors
