# Supplementary material for: Adipocytes-induced ANGPTL4/KLF4 axis drives glycolysis and metastasis in triple-negative breast cancer
Source: J Exp Clin Cancer Res. 2025 Jul 4;44:192. doi: 10.1186/s13046-025-03458-9 (PMC12231887; doi:10.1186/s13046-025-03458-9)
Supplement: Supplementary file 1 — Supplementary Material 1 [file 13046_2025_3458_MOESM1_ESM.docx]

**Supporting Information**

**Methods and Materials**

**Antibodies**

All the antibodies were purchased from Cell Signaling Technology, Abcam, Proteintech, Abclonal, HuaBio. Anti-ANGPTL4 was purchased from Abcam. Anti- PPARα, anti-β-actin, and secondary antibodies were purchased from Proteintech. Anti-Hexokinase II, anti- PFKP, anti-PGK1 were purchased from Cell Signaling Technology. Anti-PKM2, anti-LDHA were purchased from Abclonal. Anti-KLF4 was purchased from HuaBio, Alexa Fluor® 594 conjugate and goat anti-rabbit IgG (H+L) Secondary antibody was from Thermo Fisher Scientific. The recombinant human IL-6 was purchased from R&D Systems.

**Western Blotting and Real-Time qPCR**

Total protein from breast cancer cells was extracted using RIPA lysis buffer supplemented with protease and phosphatase inhibitors. Protein samples were separated by 8–12% SDS-PAGE and subsequently transferred onto a polyvinylidene fluoride (PVDF) membrane (Millipore). Following transfer, membranes were blocked with 5% bovine serum albumin (BSA) and incubated overnight at 4°C with primary antibodies diluted in 5% BSA-TBST. On the following day, membranes were incubated with the appropriate secondary antibodies for 1 hour at room temperature. Protein expression was detected using an Enhanced Chemiluminescence (ECL) detection system. The antibodies utilized in this study are listed in Additional File 2: Table 1.

Total RNA was extracted using TRIzol reagent (Vazyme, Nanjing, China) and reverse transcribed into complementary DNA (cDNA) with the HiScript QRT SuperMix for qPCR Kit (Vazyme). Quantitative real-time PCR (qRT-PCR) was performed using SYBR Green master mix (Vazyme) to quantify mRNA expression levels. The specific primers used for qPCR analysis are provided in Additional File 2: Table 1. β-actin served as an internal reference gene, and relative gene expression was calculated using the 2^−ΔΔCt^ method.

**Immunohistochemistry**

Tissue samples were excised, fixed, and embedded in paraffin, followed by sectioning into 4 μm thick slices. The paraffin-embedded sections were dewaxed using standard procedures, and antigen retrieval was conducted by heating in citrate buffer at 100°C for 2 minutes. To minimize nonspecific binding, the sections were treated with 5% BSA. Subsequently, the sections were incubated overnight at 4°C with the antibody against ANGPTL4 (1:500 dilution, Abcam). After washing with PBS, the sections were incubated with a biotin-labeled secondary antibody at room temperature for 1 hour. Immunoreactivity was detected using DAB staining, and the sections were counterstained with hematoxylin for nuclear visualization, as previously indicated.

**Immunofluorescence**

Cells were fixed with 4% paraformaldehyde at room temperature for 30 minutes and permeabilized using 0.3% Triton X-100 for 15 minutes. Nonspecific binding was minimized by blocking with 5% bovine serum albumin (BSA) for 1 hour. Primary antibody targeting KLF4 was applied, and the cells were incubated overnight at 4°C, followed by a 1-hour incubation at room temperature with a species-matched secondary antibody. Each step was separated by extensive washing to ensure removal of unbound reagents. Imaging was performed using confocal microscopy.

**EdU incorporation assay**

Cells were treated with EdU for 2 h and subsequently examined using the BeyoClick™ EdU-594 Kit (Beyotime, China) following the guidelines provided by the manufacturer. The images were captured using fluorescence microscopy.
