# Supplementary material for: Adipocytes-induced ANGPTL4/KLF4 axis drives glycolysis and metastasis in triple-negative breast cancer
Source: J Exp Clin Cancer Res. 2025 Jul 4;44:192. doi: 10.1186/s13046-025-03458-9 (PMC12231887; doi:10.1186/s13046-025-03458-9)
Supplement: Supplementary file 2 — Supplementary Material 2 [file 13046_2025_3458_MOESM2_ESM.docx]

**Tables**

**Table 1.** Sequences of the primers used to detect genes expression by qRT-PCR

| Gene name | Species | Forward Sequence | Reverse Sequence |
| --- | --- | --- | --- |
| ANGPTL4 | Human | GTCCACCGACCTCCCGTTA | CCTCATGGTCTAGGTGCTTGT |
| PPARα | Human | ATGGTGGACACGGAAAGCC | CGATGGATTGCGAAATCTCTTGG |
| HK2 | Human | TGGAGCGAGGTCTGAGCAA | ACCAGCAGGACCCGGAAAT |
| PKM2 | Human | ATGGCTGACACATTCCTGGAGC | CCTTCAACGTCTCCACTGATCG |
| LDHA | Human | GGATCTCCAACATGGCAGCCTT | AGACGGCTTTCTCCCTCTTGCT |
| PGAM1 | Human | GCTCTGCCCTTCTGGAATGAAG | ATACCAGTCGGCAGGTTCAGCT |
| β-actin | Human | CACCATTGGCAATGAGCGGTTC | AGGTCTTTGCGGATGTCCACGT |
| PGK1 | Human | CCGCTTTCATGTGGAGGAAGAAG | CTCTGTGAGCAGTGCCAAAAGC |
| PFKP | Human | CGCCTACCTCAACGTGGTG | ACCTCCAGAACGAAGGTCCTC |
| KLF4 | Human | CAGCTTCACCTATCCGATCCG | GACTCCCTGCCATAGAGGAGG |
| KLF5 | Human | CCTGGTCCAGACAAGATGTGA | GAACTGGTCTACGACTGAGGC |
| GCNT3 | Human | CACCAGAGACTGTGAGCACTTC | CATACACAGCTCGCAGTAGCCT |
| SOCS3 | Human | CCTGCGCCTCAAGACCTTC | GTCACTGCGCTCCAGTAGAA |
| SNAIL1 | Human | TCGGAAGCCTAACTACAGCGA | AGATGAGCATTGGCAGCGAG |
| PRTN3 | Human | AACTACGACGCGGAGAACAAA | CGAGGGACGAAAGTGCAAATG |
| WFDC3 | Human | TCGGATCTGCCGAGACATTCCT | CTACACAGCTCTTGTTGCAGCC |
| VNN1 | Human | GACCCCAGTACAAGAAAGACTCA | TCAGGCTCCTTGGGTACATTG |
| LOXL2 | Human | GGGTGGAGGTGTACTATGATGG | CTTGCCGTAGGAGGAGCTG |
| IL-1RL1 | Human | CTCTGTTTCCAGTAATCGGAGCC | GCAGCCAAGAACTGAGTGCCTT |
| FOSL1 | Human | CAGGCGGAGACTGACAAACTG | TCCTTCCGGGATTTTGCAGAT |
| SIRT1 | Human | TAGACACGCTGGAACAGGTTGC | CTCCTCGTACAGCTTCACAGTC |
| PPF1A4 | Human | CTCTGCGGATGTTGTCTCCC | ATGCTGCCACTGGTTACACG |
| NDRG1 | Human | CTCCTGCAAGAGTTTGATGTCC | TCATGCCGATGTCATGGTAGG |
| SPAG4 | Human | TCTCCAGTAGTCTCTGAGGAGC | CGGATGGAACAGACCTCCC |
| NNMT | Human | ATATTCTGCCTAGACGGTGTGA | TCAGTGACGACGATCTCCTTAAA |
| FGG | Human | TTATTGTCCAACTACCTGTGGC | GACTTCAAAGTAGCAGCGTCTAT |
| ENGL3 | Human | CTGGGCAAATACTACGTCAAGG | GACCATCACCGTTGGGGTT |

**Table 2.** Correlation between ANGPTL4 expression and clinicopathological characteristics in breast cancer patients

|  | variables | ANGPTL4 expression | | total | χ2 | p value |
| --- | --- | --- | --- | --- | --- | --- |
|  |  | low | High |  |  |  |
| Age (year) |  |  |  |  | 1.227 | 0.268 |
|  | < 55 | 12 | 17 | 29 |  |  |
|  | >= 55 | 8 | 21 | 28 |  |  |
| Grade |  |  |  |  | 0.07 | 0.791 |
|  | II | 16 | 16 | 32 |  |  |
|  | III | 13 | 12 | 25 |  |  |
| T stage |  |  |  |  | 0.468 | 0.494 |
|  | T1 | 15 | 16 | 31 |  |  |
|  | T2 -T3 | 14 | 12 | 26 |  |  |
| M stage |  |  |  |  | 3.331 | 0.068 |
|  | M0 | 21 | 18 | 39 |  |  |
|  | M1 | 4 | 11 | 15 |  |  |
| N stage |  |  |  |  | 0.300 | 0.584 |
|  | N0 | 14 | 16 | 30 |  |  |
|  | N1-3 | 13 | 11 | 24 |  |  |
| TNM stage |  |  |  |  | 8.361 | 0.039 |
|  | TNM I-II | 20 | 16 | 36 |  |  |
|  | TNM III-IV | 8 | 13 | 21 |  |  |
| ER |  |  |  |  | 0.285 | 0.594 |
|  | negative | 13 | 15 | 28 |  |  |
|  | positive | 11 | 18 | 29 |  |  |
| PR |  |  |  |  | 1.733 | 0.188 |
|  | negative | 17 | 12 | 29 |  |  |
|  | positive | 11 | 17 | 28 |  |  |
| HER2 |  |  |  |  | 0.072 | 0.788 |
|  | negative | 18 | 16 | 34 |  |  |
|  | positive | 11 | 12 | 23 |  |  |
| KI67 |  |  |  |  | 0.132 | 0.717 |
|  | negative | 4 | 5 | 9 |  |  |
|  | positive | 25 | 24 | 49 |  |  |
| Tumor size  >=2cm |  |  |  |  | 0.306 | 0.580 |
|  | negative | 11 | 18 | 29 |  |  |
|  | positive | 18 | 20 | 28 |  |  |
|  |  |  |  |  |  |  |

* Statistically significant(p<0.05)
