# Supplementary material for: Adipocytes-induced ANGPTL4/KLF4 axis drives glycolysis and metastasis in triple-negative breast cancer
Source: J Exp Clin Cancer Res. 2025 Jul 4;44:192. doi: 10.1186/s13046-025-03458-9 (PMC12231887; doi:10.1186/s13046-025-03458-9)
Supplement: Supplementary file 3 — Supplementary Material 3 [file 13046_2025_3458_MOESM3_ESM.docx]

**Supplementary Figures**


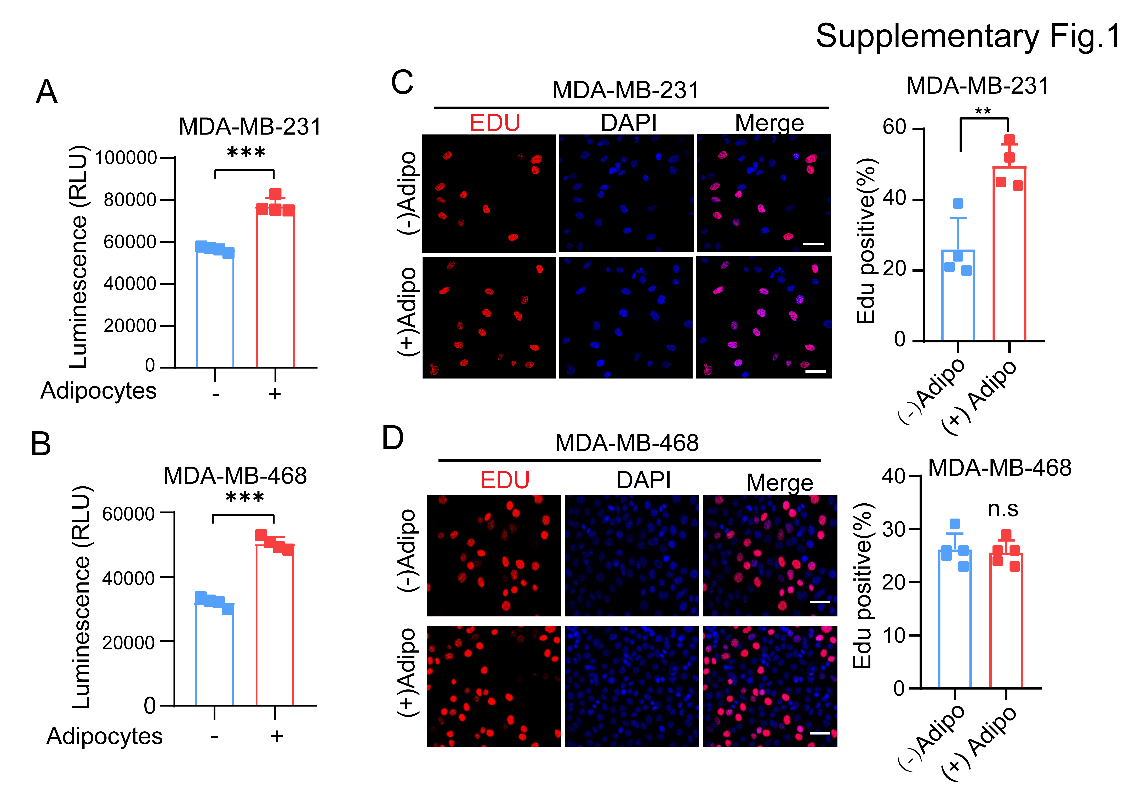


**Supplemenary Fig.1 Effects of adipocytes on ATP production and proliferation in TNBC cells.** (A-B). ATP production was assessed in TNBC cells, both with and without coculturing with adipocytes (n= 4). (C-D). EdU incorporation assay was employed to measure the contribution of adipocytes on proliferation of TNBC cells (n=4 or 5). Scale, 20μm. *p<0.05; **p<0.01; ***p<0.001.


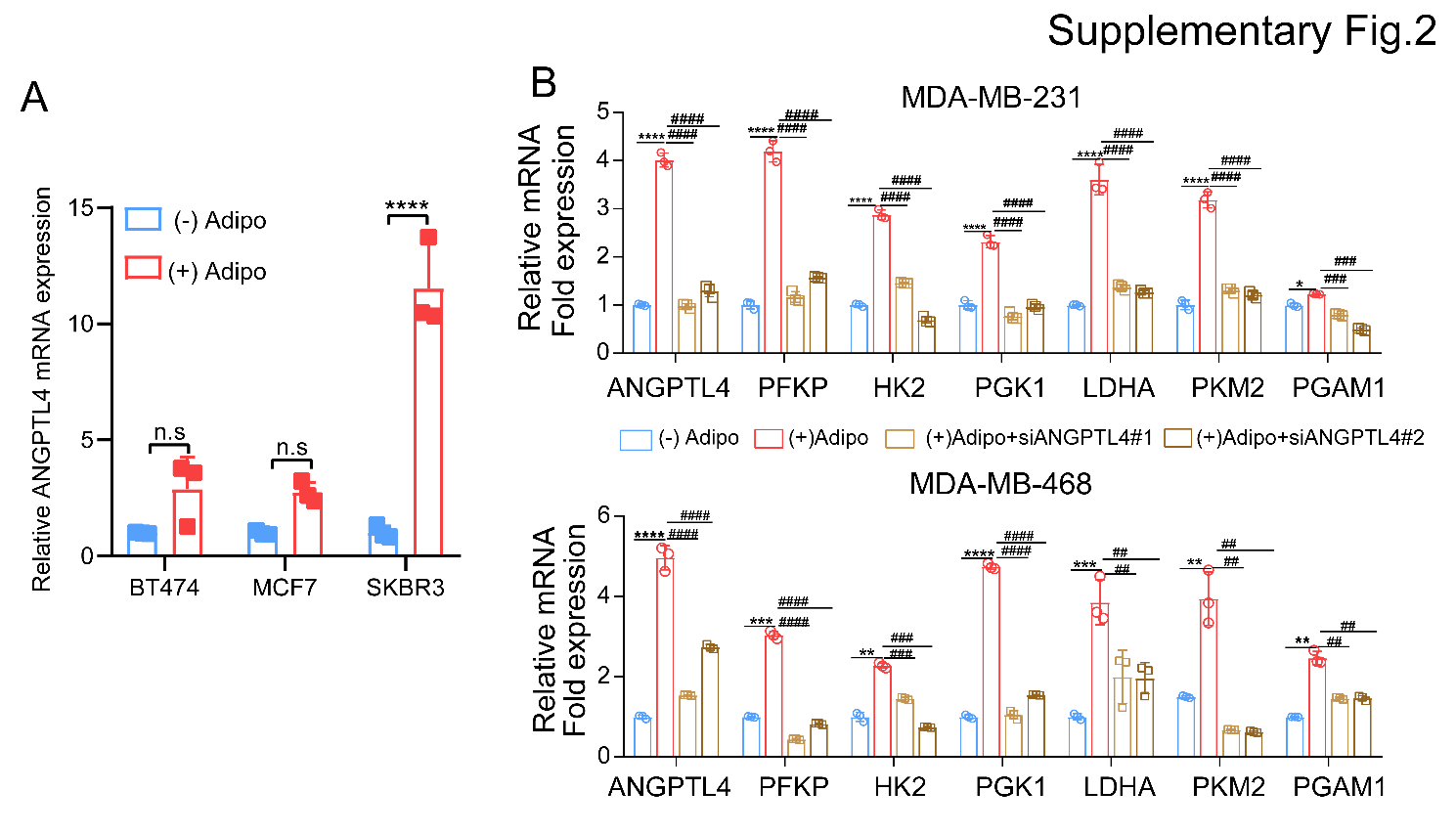
 **Supplemenary Fig.2 Effects of ANGPTL4 on adipocytes-induced glycolysis in TNBC cells.** (A). qPCR analysis to detect the mRNA levels of ANGPTL4 in different breast cancer cells in the presence or absence of adipocytes. (B). qPCR analysis to assess the mRNA levels of ANGPTL4, HK2, PFKP, PGK1, PKM2, LDHA, PGAM1 expression in TNBC cells in cocultured TNBC or monoculture TNBC cells. * coculture vs. monoculture, *p<0.05; **p<0.01; ***p<0.001, # coculture with siANGPTL4 vs. coculture, #p<0.05; ##p<0.01; ###p<0.001, ####p<0.0001.


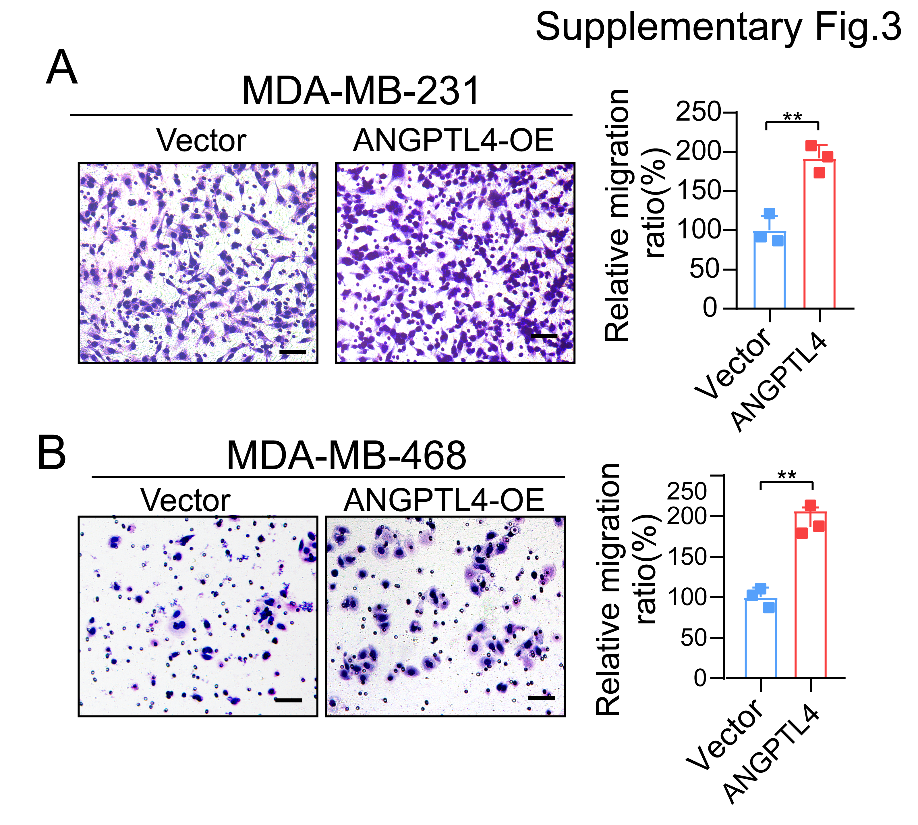


**Supplemenary Fig.3 The role of ANGPTL4 on the migration ability of TNBC cells.** (A-B). Transwell assay to assess the role of ANGPTL4 on the migration ability of TNBC cells. Scale bar 100 µm. *p<0.05; **p<0.01.


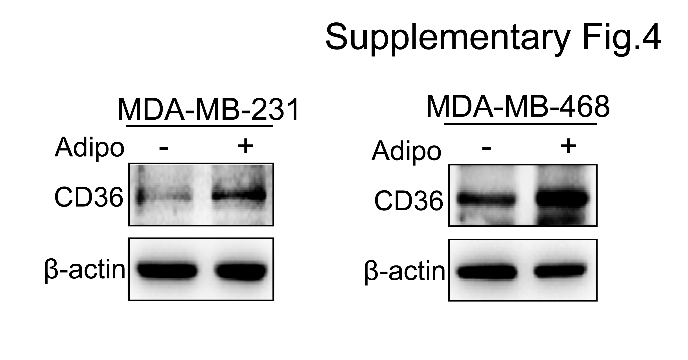


**Supplemenary Fig.4 Effects of adipocytes on CD36 expression in TNBC cells.** Western blot analysis to assess CD36 in cocultured TNBC cells or monoculture TNBC cells.


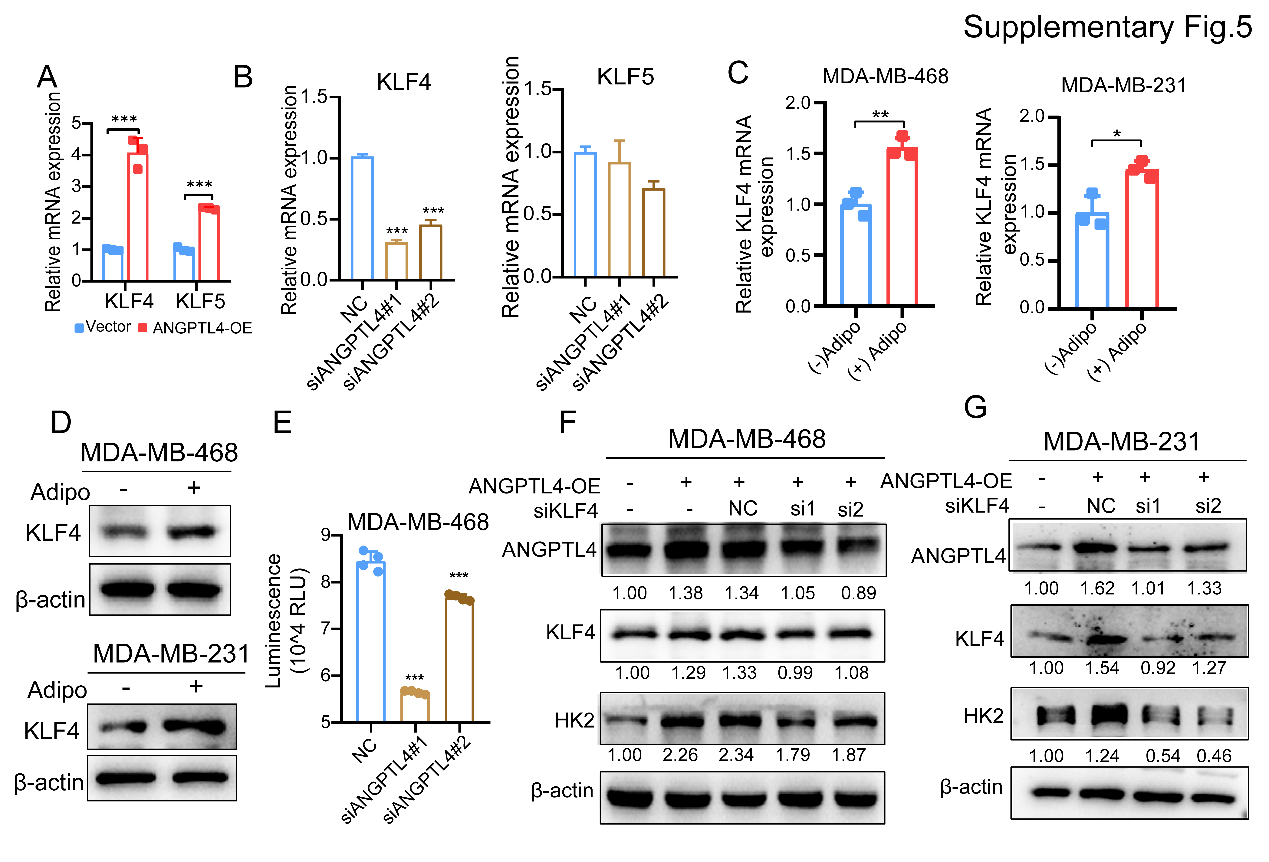


**Supplemenary Fig.5 The effect of KLF4 on ANGPTL4 induced glycolysis in TNBC cells.** (A-B) qPCR analysis to detect the mRNA levels of KLF4 and KLF5 in TNBC cells with ANGPTL4 ectopic expression or ANGPTL4 knockdown. (C) qPCR analysis to detect the mRNA levels of KLF4 in TNBC cells in the presence or absence of adipocytes. (D) Western blot analysis to measure KLF4 expression in cocultured TNBC cells or monoculture TNBC cells. *p<0.05; **p<0.01. (E) Total ATP production in TNBC cells with ANGPTL4 knockdown (n= 4). (F-G) Western blot analysis of KLF4, ANGPTL4 and glycolytic-related enzymes in MDA-MB-468 and MDA-MB-231 cells after ANGPTL4 overexpression or overexpression of ANGPTL4 accompanied with KLF4 knockdown.


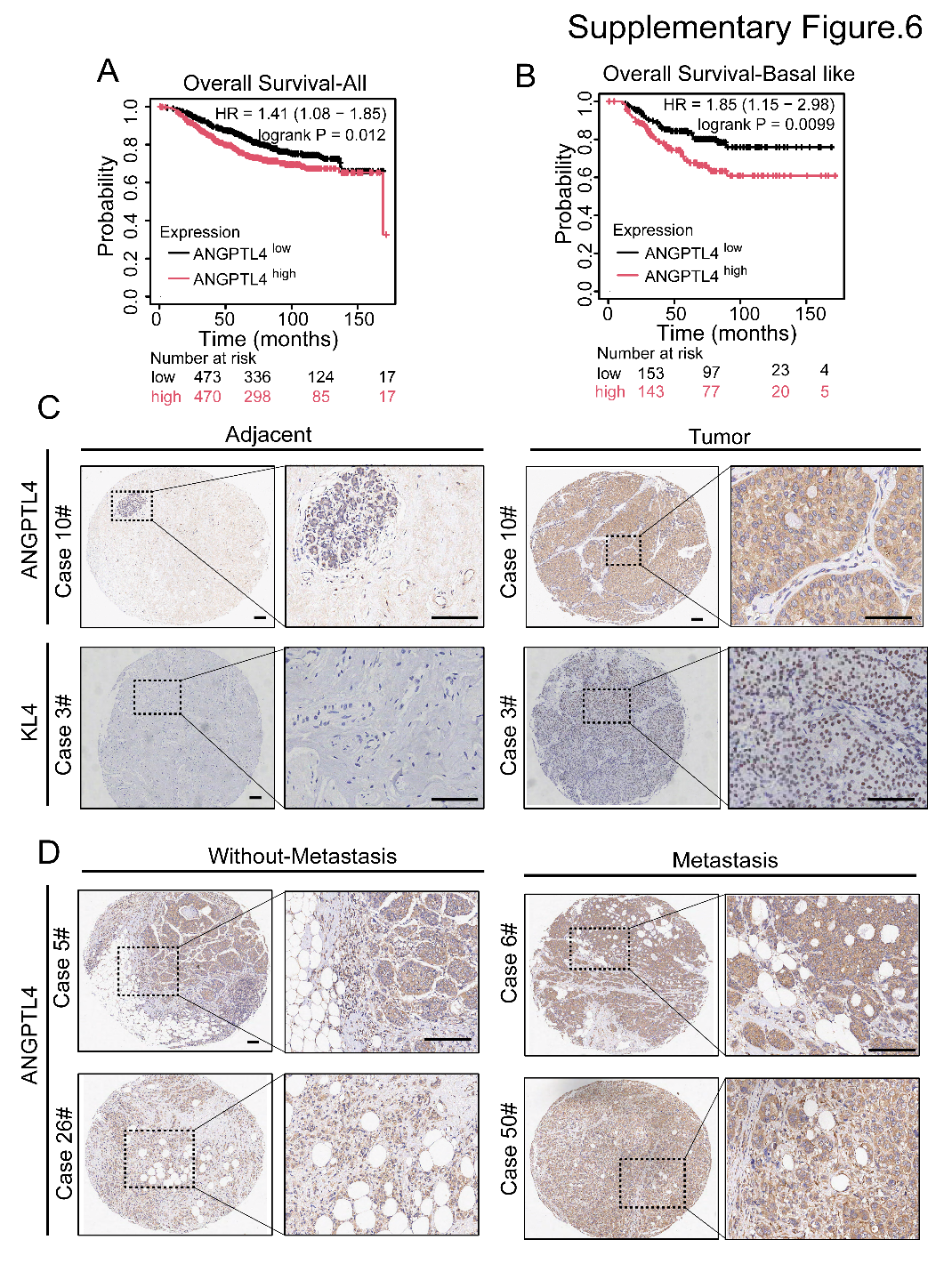


**Supplemenary Fig.6 ANGPTL4 and KLF4 is associated with prognosis in patients with breast cancer.** (A-B) Kaplan-Meier analysis of Overall survival - All (OS) and Overall survival -Basal like in breast cancer patients. ANGPTL4 in all breast cancer patients (n = 943) and in basal-like breast cancer patients (n = 296). (C-D) The representative of IHC for ANGPTL4 and KLF4 expression in breast cancer tissue and adjacent tissue. adjacent tissue refers to the tissue far than 2 cm away from the tumor foci.
